# Differential early response of monocyte/macrophage subsets to intra-operative corticosteroid administration in lung transplantation

**DOI:** 10.3389/fimmu.2023.1281546

**Published:** 2023-10-24

**Authors:** Matthieu Glorion, Florentina Pascale, Maxime Huriet, Jérôme Estephan, Carla Gouin, Céline Urien, Mickael Bourge, Giorgia Egidy, Christophe Richard, Valérie Gelin, Julien De Wolf, Morgan Le Guen, Antoine Magnan, Antoine Roux, Philippe Devillier, Isabelle Schwartz-Cornil, Edouard Sage

**Affiliations:** ^1^ Department of Thoracic Surgery and Lung Transplantation, Foch Hospital, Suresnes, France; ^2^ Université Paris-Saclay, INRAE, UVSQ, VIM, Jouy-en-Josas, France; ^3^ Cytometry/Electronic Microscopy/Light Microcopy Facility, Imagerie-Gif, Université Paris-Saclay, CEA, CNRS, Institute for Integrative Biology of the Cell (I2BC), Gif-sur-Yvette, France; ^4^ Université Paris-Saclay, INRAE, AgroParisTech, GABI, Jouy-en-Josas, France; ^5^ Université Paris-Saclay, UVSQ, INRAE, BREED, Jouy-en-Josas, France; ^6^ Department of Anesthesiology, Foch Hospital, Suresnes, France; ^7^ Department of Pulmonology, Foch Hospital, Suresnes, France; ^8^ Respiratory Pharmacology Research Unit - Exhalomics, Foch Hospital, Suresnes, France

**Keywords:** lung, transplantation, pig model, monocytes-macrophages, corticosteroids, ischemia-reperfusion section: allo-immunity and transplantation

## Abstract

**Introduction:**

Lung transplantation often results in primary and/or chronic dysfunctions that are related to early perioperative innate allo-responses where myeloid subsets play a major role. Corticosteroids are administered upon surgery as a standard-of-care but their action on the different myeloid cell subsets in that context is not known.

**Methods:**

To address this issue, we used a cross-circulatory platform perfusing an extracorporeal lung coupled to cell mapping in the pig model, that enabled us to study the recruited cells in the allogeneic lung over 10 hours.

**Results:**

Myeloid cells, i.e. granulocytes and monocytic cells including classical CD14^pos^ and non-classical/intermediate CD16^pos^ cells, were the dominantly recruited subsets, with the latter upregulating the membrane expression of MHC class II and CD80/86 molecules. Whereas corticosteroids did not reduce the different cell subset recruitment, they potently dampened the MHC class II and CD80/86 expression on monocytic cells and not on alveolar macrophages. Besides, corticosteroids induced a temporary and partial anti-inflammatory gene profile depending on cytokines and monocyte/macrophage subsets.

**Discussion:**

This work documents the baseline effects of the standard-of-care corticosteroid treatment for early innate allo-responses. These insights will enable further optimization and improvement of lung transplantation outcomes.

## 1 Introduction

Allogeneic lung transplantation (LT) is the ultimate therapeutic option for end-stage respiratory diseases. However, the median survival time is only approximately six years due to frequent complications, i.e. primary graft dysfunction (PGD), chronic allograft lung dysfunction, infections, and the secondary effects of immunosuppressive drugs (1). A shift of paradigm is required to improve the LT outcome and reduce the burden of long-term immunosuppressive drug treatments.

It has recently been proposed that the control of the innate immune responses during LT, through targeting myeloid cells, could result in tolerance induction and prolonged graft survival (2, 3). The innate immune responses in LT are induced by the ischemia-reperfusion process that inevitably occurs upon reoxygenation of the explanted lung and is responsible for a complex sterile inflammatory cascade driven by polymorphonuclear cells (PMNs), resident alveolar macrophages (AMs) and monocytic cell subsets of hematopoietic origin (4). Monocytic cells (MoCs) encompass monocytes, monocyte-derived macrophages, and dendritic cells (DCs), that are characterized by different activation, tissue-location, and differentiation statuses (5). Monocytes are distinguished into classical (CD14^pos^) and non-classical/intermediate (CD16^pos^) monocytes in humans (6), with similar counterparts in mice (7) and in other mammals such as pigs (8). In mouse models these subsets have been shown to play distinct roles in the innate response to ischemia-reperfusion upon LT in an allogeneic context: in the first hours post-LT, the donor non-classical monocytes initiate the PMN recruitment, and the recipient classical monocytes promote the PMN extravasation which is necessary to PGD, both through IL-1β signaling (4). Experimental data also support that the combination of ischemia-reperfusion and innate allogeneic recognition signaling activates the recipient monocytes that differentiate either into pulmonary macrophages or inflammatory DCs and trigger allogeneic T and B cell responses leading to chronic rejection (9, 10). At the clinical level, the severity of ischemia-reperfusion injury is related to PGD development in the first three days post-LT (11) and PGD has been associated with later rejection and chronic lung allograft dysfunction (12). Altogether, the current knowledge supports that the innate myeloid response that occurs in the first hours post-LT is the driving mechanism that, through uncontrolled amplification, leads to PGD.

In the clinic of LT, methylprednisolone (i.e. corticosteroids, CS) is the cornerstone of early immunosuppressive therapy. Methylprednisolone is given intraoperatively as a bolus before reperfusion (13, 14), however, its effects on the detrimental early innate myeloid responses described above are not known. Indeed, while the inhibitory effects of CS on adaptative immunity, particularly on T-cells are well described, their effect on the innate myeloid cells appears complex and may depend on subsets and their differentiation and activation stage (15). Furthermore, most knowledge about the CS effects was obtained from *in vitro* cultured monocytes or macrophages (15, 16) and may not recapitulate the *in vivo* response, in particular in the context of innate allo-responses of LT. It is therefore of prime importance to dissect the effects of current treatments given at the surgical time on the innate allo-responses during the first hours post-LT, to develop needed improvements.

The pig animal model is relevant to the study of human LT due to the anatomical, physiological, and immunological similarities between pigs and humans, which are much closer than between rodents and humans (17). Furthermore, rodents are not considered reliable preclinical models when examining immunomodulatory drugs in transplantation (18). We recently established a cross-circulation platform of extracorporeal donor lung coupled to cell mapping in the pig (19). We showed that this model presents several advantages over classical LT to study the cellular and molecular events occurring upon ischemia-reperfusion and the first encounter between donor and recipient cells. Indeed in that model (**Supplemental Figure 1**), an extracorporeal donor lung is connected to the circulation of a perfusing pig via the superior vena cava, through a rapid and easy procedure that is ethically friendly, which permits a tight control of the vascular flow and the ventilation and thus leads to a high degree of experimental repeatability leading to robust results across experiments. Furthermore, a longitudinal sampling can be easily performed from an accessible extracorporeal lung at different times. Finally, an intravenous injection of CFSE that labels the whole leukocyte compartment of the perfusing pig allows to distinguish the pig donor cells from the perfusing pig cells and to take into account the regulatory systemic signaling of a complete host, which would not be the case with a classical *ex vivo* lung perfusion (EVLP) using whole blood. Thanks to this model, we showed that PMNs and MoCs are the dominantly recruited populations from the pig host in the first 10 h and that the recruited MoCs upregulate the MHC class II and CD80/86 antigen-presentation molecules at their cell surface (19). Here we used this cross-circulatory platform to study the effect of a CS pulse -similar to the one given upon surgery- on the innate allogeneic cell activation, and in particular on AMs and recruited classical (CD14^pos^) and non-classical/intermediate (CD16^pos^) MoCs, the latter upregulating MHC class II and CD80/86 upon reperfusion. We uncovered that CS did not modify the innate cell recruitment, they dampened the expression of MHC class II and CD80/86 on MoCs and not on AMs, and partially and temporally reduced inflammatory cytokine gene expression depending on cytokine genes and monocyte/macrophage subsets.

## 2 Materials and methods

### 2.1 Animals

Large-White pigs (ten donor pigs and ten perfusing pigs) were hosted in the Animal Genetics and Integrative Biology unit (GABI-INRAE, France). We used matched pairs of male donors and female recipients (i.e. perfusing pigs) from different siblings. Pigs were 3 - 5 months old, with a mean weight of 50.8 ± 4.4 kg in the untreated (UT) group and 49.9 ± 1.8 kg in the CS treated (CST) group.

### 2.2 Donor lung harvest and lung cannulation

The lung harvest from donor pigs (*n* = 10) was performed in the Animal Surgery and Medical Imaging Platform (CIMA-MIMA2-BREED-INRAE, Jouy en Josas, France) as described in a previous study (19). The ischemic durations undergone by the donor lungs are provided in **Supplemental Table 1** for the UT and CST groups (warm ischemia duration: 88 ± 5.7 min for UT and 79.6 ± 11.8 min for CST group, cold ischemia duration: 107.6 ± 18.6 min for UT and 100.2 ± 12.85 min for CST group).

### 2.3 Recipient conditioning and cross-circulation

The overall cross-circulation procedure is depicted in **Supplemental Figure 1**. Recipient pigs (*n* = 10) were anesthetized as described (19). Septotryl® (0.08 ml/kg) (Vetoquinol) was injected i.m. before catheterization. In the CST group, 20 mg/kg methylprednisolone (Mylan, Canonsburg, USA) was administered i.v. 1 h prior to the start of cross circulation. After a 25,000 U heparin bolus, the recipient pig was maintained on a continuous heparin infusion (100 U/Kg/h). The superior vena cava was cannulated in the recipient pig with a 20 F double lumen cannula as described (19). Carboxyfluorescein succinimidyl ester (CFSE, 25 mg, Sigma-Aldrich, Saint-Louis, MS, USA) was administered i.v. in the perfusing pig, diluted in 4 ml DMSO + 40 µl heparin, 30 min before the initiation of cross-circulation. Donor lungs were placed in dorsal position on XVIVO® chambers (XVIVO Perfusion) and the trachea was cannulated with a 7.5 mm diameter cuffed endotracheal tube (Mallinckrodt, Staines-upon-Thames, UK) and connected to a respirator. The vascular tubing was spliced to connect the recipient pig to the dedicated circuit, marking the start of cross-circulation. Sedation was kept for 10 h by permanent administration of 2 mg/kg/h propofol (Proposure®, Axience, Pantin, France) with 0.6% isofluorane and analgesia was achieved by i.v. administration of 0.2 mg/kg nalbuphine every 3 hours.

### 2.4 Extracorporeal haemodynamic and lung function monitoring

We collected blood samples from the PA and PV cannula hourly to perform the hemo-gas analyses using an Istat® kit. The static compliance (Tidal Volume/(plate pressure – positive end expiratory pressure)), ΔPCO2 (arterial PCO2 – venous PCO2) and ΔPO2/FIO2 (venous PO2 – arterial PO2/FIO2) were measured every 1 h. The transpulmonary pressure (PA - PV) and the pulmonary vascular resistance were calculated (PA pressure – left atrial pressure) x 80/flow rate.

### 2.5 Blood sample collection

We collected blood samples by venipuncture of the auricular vein or directly from the extracorporeal circuit. Blood counts and biochemical profiling from plasma were performed on an MS4.5 analyzer and an M-Scan II analyzer (Melet Schloesing Laboratoires, Cergy-Pontoise, France).

### 2.6 Lung biopsies

Lung biopsies for cell dissociation (about 2 g) were sampled from the cranial and caudal lobes using a surgical stapler (Endo GIA™ universal stapling system, Medtronic, Minneapolis, USA) and were immediately immerged in cold hypothermic preservation media (HypoThermosol® FRS, Stemcell Technologies Inc, Vancouver, Canada). For histology, biopsies were fixed in cold phosphate-buffered 4% paraformaldehyde for 24 h and subsequently paraffin-embedded.

### 2.7 Lung cell extractions

Lung tissue (2 g) was finely chopped and incubated at 37°C for 45 minutes under rotation in RPMI 1640 supplemented with 100 IU/ml penicillin,100 μg/ml streptomycin, 2 mM L-glutamine and 10% inactivated fetal calf serum (FCS) (Invitrogen, Paisley, UK), containing 3 mg/ml collagenase D, 0.25 mg/ml DNase I (Sigma-Aldrich) and 0.7 mg/ml dispase II (Gibco®, ThermoFisher Scientific, St Aubin, France). The cell preparation was subsequently filtered on a nylon mesh (1 mm diameter) followed by successive passages through cell strainers (500 µm, 100 µm, 40 µm). Erythrocytes were eliminated with erythrocyte lysis buffer (10 mM NaHCO3, 155 mM NH4Cl, and 10 mM EDTA). The cells were washed in PBS, counted and 10^8^ cells were processed to cell surface staining. For subsequent use (cell sorting), the remaining cells were frozen in 10% DMSO + 90% FCS using a Mister Frosty freezing container (Nalgene, Rochester, NY, USA) and finally kept in liquid N2.

### 2.8 Cell surface staining and flow cytometry strategy

We performed the cell surface staining in RPMI supplemented with 10 mM Hepes, and 5% horse and swine serum respectively (Gibco, Life Technologies Europe, Bleiswijk, Netherlands). We used swine-specific primary antibodies (Abs) and conjugated secondary Abs that are listed in **Supplemental Table 2**. We used isotype controls (mouse IgG1, IgG2b, and IgG2a) at the same concentration as the tested mAbs, based on the fluorescence minus one method (20). In some cases, a directly PE-conjugated mAb (anti-CD163-PE and anti-CD3-PE), of the same IgG1 isotype as a primary Ab, was used as a third step, and in these cases, an additional saturation step was done with an excess of mouse IgG1 (50 μg/ml). A DAPI staining (Sigma-Aldrich) excluded dead cells. Results were acquired on BD LSR Fortessa™ Cell Analyzer (BD-Biosciences). The FACS data were analyzed with the FlowJo software (version 10.7.1; Tree Star, Ashland, OR, USA). The gating strategy is shown in **Supplemental Figure 2** which originates from our recent paper (19).

### 2.9 Cell sorting of AMs, CD14^+,^ and CD16^+^ MoCs

Lung cells were thawed from frozen stocks and reacted with anti-CD14, CD16, and CD172A mAbs followed by conjugated goat anti-mouse isotype-specific Abs (**Supplemental Table 2**). The anti-IgG1 conjugate was saturated by an excess of mouse IgG1 (50 μg/ml) and cells were labeled with anti-CD163-PE (IgG1) (**Supplemental Table 2**). The cell subsets were sorted (“purity” mode) by flow cytometry on the Imagerie-Gif Cytometry facility (I2BC, Gif sur Yvette, France) using the MoFlo ASTRIOS sorter (Beckman-Coulter, Paris, France) and the Summit 5.2 software. AMs were sorted as CFSE^neg^SSC^hi^CD163^hi^/CD172A^hi^ from total live lung cells (see (19) for their characterization). The MoCs were sorted as CFSE^pos^SSC^lo^CD172A^hi^CD14^pos^CD16^lo^ (named CD14^pos^) and CFSE^pos^SSC^lo^CD172A^hi^CD14^lo^CD16^hi^ cells (named CD16^pos^).

### 2.10 RNA extraction, reverse-transcription, and RT-qPCR

Total mRNA from sorted CD14^pos^, CD16^pos^, and AMs as well as RNA from total lung cells used as a calibrator were extracted using the Arcturus (PicoPure™ RNA kit-ThermoFisher Scientific) and quantified by Qubit™ RNA high sensitivity kit (Invitrogen™, Fisher Scientific SAS, Illkirch, France). RNA was reverse-transcribed using random primers and the Multiscribe reverse transcriptase (Applied Biosystem, ThermoFisher Scientific), using equal starting quantities of RNA from test and calibrator RNA (8 to 50 ng). Quantitative real-time PCR was carried out with 300 nM primers in a final reaction volume of 25 µl of 1 X SYBR Green PCR Master Mix (Applied Biosystem, ThermoFisher Scientific). The primers were designed using the primer express software (v2.0) and are reported in **Supplemental Table 3**. PCR cycling conditions were 95°C for 30 sec, linked to 40 cycles of 95°C for 5 sec and 60°C for 30 sec. Real-time qPCR data were collected by the Bio-Rad CFX Maestro system (Bio-Rad Laboratories Inc, Marne-la-Coquette, France) and expression of the different genes relative to RPS24 and normalized to the internal calibrator (arbitrary units) were calculated by the 2^-ΔΔCt^ method.

### 2.11 May–Grunwald–Giemsa staining and histology

Sorted cells were spun onto microscope slides by cytocentrifugation and stained with May–Grunwald–Giemsa stain. Images were acquired with a Leica Leitz DMRB microscope equipped with a 63 oil-immersion objective (numerical aperture 1.3), an ExiAquaTM imaging camera, and the QCapture software (QImaging, Surrey, Canada). Formalin-fixed paraffin-embedded lung tissues from a UT and CST experiment at 0 h, 6 h, and 10 h were cut every 50 µm to produce three 5 µm tissue slices per sample that were stained with hematoxylin-eosin-saffron (HES). The slides were imaged with a slide scanner (Pannoramic SCAN II, v3.0.2, 3DHistech, Medipixel Ltd, Budapest, Hungary) and were observed by an external pathologist and a veterinarian in a blinded manner. Five randomly-selected high power fields per slide (7x10^4^ µm^2^ area) were observed from 3 slides per sample originating from 2 pigs per group (leading to 6 slide results per timing in each group) and the airway and alveolar polymorphonuclear cells were quantified.

### 2.12 Statistics

Data were analyzed with the GraphPad Prism 8.0 software. After subjecting the data to a normality test, a paired parametric two-tailed t-test was used to compare values between different time points, and an unpaired parametric two-tailed t-test was used to compare values between different treatments (UT and CST). When the data did not pass the normal distribution test, a non-parametric paired Wilcoxon signed rank test was used for analyzing differences between timing (paired data), and a non-parametric Mann-Whitney test was used for analyzing differences between UT and CST groups (unpaired data). Mean ± standard deviations were calculated.

### 2.13 Study approval

The animal experiments were conducted in accordance with EU guidelines and French regulations (DIRECTIVE 2010/63/EU, 2010; Code rural, 2018; Décret n°2013-118, 2013). The experiments were approved by the COMETHEA ethic committee under the APAFIS number authorization 25174-2020011414322379 and were authorized by the French “Ministère de l’enseignement supérieur et de la recherche”.

## 3 Results

### 3.1 Recruited and donor monocytic cells in lung grafts include CD14^pos^ and CD16^pos^ subsets with upregulation of MHC class II and CD80/86 expression in the CD16^pos^ subset upon allogeneic reperfusion

**Figure 1:**
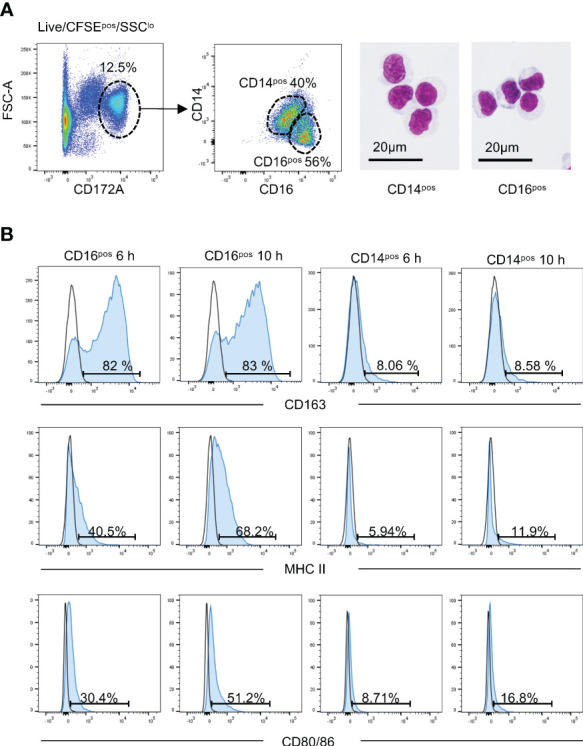
The recruited monocytic cells in lung grafts include CD16^pos^ and CD14^pos^ subsets. **(A)** The live CFSE^pos^SSC-A^lo^ cells were selected as we reported in (19). The CD172A^hi^ cells were gated and were further split into CD14^pos^ and CD16^pos^ cells. The two subsets were sorted by flow cytometry and stained with May-Grunwald Giemsa. The represented gating strategy is shown on a pig cross-circulation experiment at 10 h, depicted as a cross symbol in other figures. **(B)** The expression of CD163, MHC class II, and CD80/86 on the CD16^pos^ and CD14^pos^ cells of the live CFSE^pos^SSC-A^lo^CD172A^hi^ lung cells is depicted (filled blue histogram) versus an IgG2a isotype control (black line) at 6 h and 10 h post-reperfusion. The percent of positive cells are indicated.

Results from the mouse model of lung transplantation indicate that the cell recruitment and activation that occur in the first hours following LT, play key roles in the ischemia reperfusion injury and PGD development (4). To study these early events in an animal model of high translational value, i.e. the pig model, we used the cross-circulation platform coupled with cell mapping that we presented in the introduction (19). We monitored the recruitment of the host immune cells that perfuse an extracorporeal allogeneic lung over 10 h, a pertinent time frame based on the murine results (4). In particular, we analyzed the composition of the recruited MoCs cells in the CD14^pos^ (classical) and CD16^pos^ (non-classical/intermediate) subsets. CFSE was injected intravenously into the perfusing pig just before the cross-circulation initiation, resulting in the CFSE labeling of most circulating host cells (see Material and Methods, and (19)). Host MoCs cells in the graft were identified as live CFSE^pos^SSC^lo^CD172A^hi^ cells, as reported in (19) and shown in **Supplemental Figure 2**, and they included 38.2 ± 16.9% CD14^pos^ MoCs and 57.3 ± 5.4% CD16^pos^ MoCs at 6 h post cross-circulation initiation (**Figure 1A**, **Supplemental Figure 3A**). May-Gründwald staining of these two subsets revealed a monocyte/macrophage morphology with a bean shaped nucleus, relatively loose chromatin, and large cytoplasm indicative of cell activation (**Figure 1A**). These cells might be engaged in a macrophage differentiation but our characterization method did not allow us to conclude on their differentiation status. We therefore designate the recruited CFSE^pos^SSC^lo^CD172A^hi^ as MoCs. The recruited CD16^pos^ subset and not the CD14^pos^ subset expressed high levels of the scavenger molecule CD163, consistent with their non-classical/intermediate identity (8, 21) (**Figure 1B**). The proportions of the recruited CD14^pos^ and CD16^pos^ subsets remained stable over 10 h cross-circulation (**Supplemental Figure 3A**).

At T0 h, the CFSE^neg^SSC^lo^CD172A^hi^ cells, which correspond to donor cells, also included CD14^pos^ (20.9 ± 6.7%) and CD16^pos^ (69.4 ± 5.7%) subsets (**Supplemental Figure 3B**, **Supplemental Figure 4**). At 6 h and 10 h, the proportions of CD14^pos^ and CD16^pos^ cells in the CFSE^neg^SSC^lo^CD172A^hi^ cells were not modified when compared to 0 h (**Supplemental Figure 3B**). Note that the CFSE^neg^SSC^lo^CD172A^hi^ cells during reperfusion may also encompass some lowly CFSE-labeled cells originating from the perfusing pig (19).

We found previously that the recruited MoCs during the cross-circulation upregulated between 6 h and 10 h the surface expression of MHC class II and CD80/86, which are antigen presentation molecules that play a major role in allo-stimulation and rejection (19). Interestingly **Figure 1B** shows that the upregulation of MHC class II and CD80/86 dominantly concerned the recruited CD16^pos^ subset and increased between 6 h and 10 h. The expression of these antigen-presentation molecules was also confined to the CD16^pos^ subset within the CFSE^neg^ cells (**Supplemental Figure 4B**). 

Overall the allogeneic reperfusion of lung graft implicates the recruitment of both CD14^pos^ and CD16^pos^ subsets and leads to the upregulation of MHC class II and CD80/86 on the CD16^pos^ subset on the donor and perfusing pig cells.

### 3.2 The corticosteroid treatment does not modify the composition of the early recruited cell subsets in lung graft

Corticosteroids (CS) in the form of methylprednisolone are given intraoperatively as a bolus at a dose of 1 g per patient before the reperfusion (13) and thereafter, corticosteroids and/or other immunosuppressive drugs are administered starting from the next day. In order to assess the effect of the intraoperative treatment with CS on the early innate allogeneic response in lung graft, we administered methylprednisolone i.v. 1 h before cross-circulation initiation in the perfusing pig, at a “bolus” dose classically used in pig lung transplantation (i.e. 20 mg/kg (22–24),), which is in the same order of magnitude as the dose used in humans. We followed the clinical parameters hourly and performed tissue sampling at 0 h (before cross-circulation initiation), 6 h and 10 h.

The static compliance, ΔPO2:FiO2, and pulmonary vascular resistance remained stable in the graft throughout the whole procedure with no statistically significant difference between the untreated (UT) and CS-treated (CST) groups (**Supplemental Figures 5A-D**). In the perfusing pigs, vital parameters (heartbeats, blood pressure, lactate, creatinine, glucose levels) lay within normal values (**Supplemental Table 4**) and blood counts were within the normal range (**Supplemental Table 4**). Cell viability was measured with the exclusion of DAPI on dissociated lung cell preparations and was maintained over time, independently of CS administration (**Supplemental Figure 5E**). The airway and parenchymal structures were preserved over time, showing no sign of oedema, no sign of injury, and a weak infiltration with inflammatory polymorphonuclear cells (PMNs) that increased in both groups between timings (**Supplemental Figure 6**, p < 0.05). 

**Figure 2:**
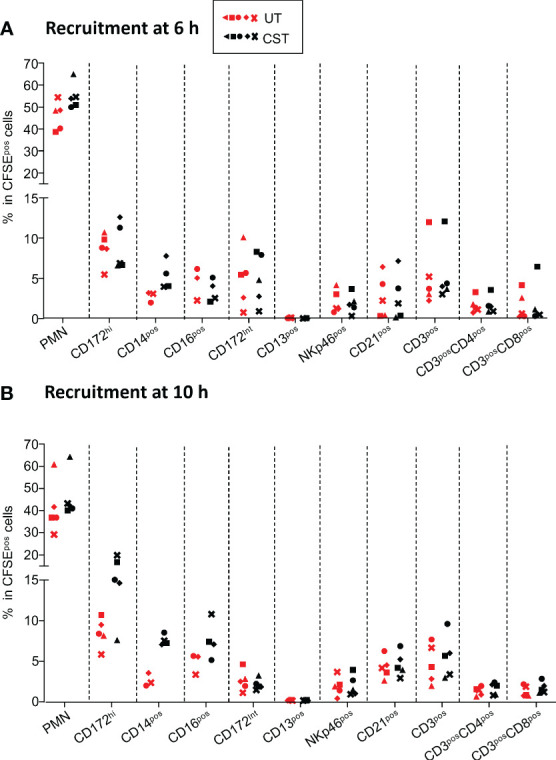
Representation of immune cell subsets among the CFSE^pos^ cells at 6 h **(A)** and 10 h **(B)** in lung grafts. The gating strategy has been described in (19) and is also shown **Supplemental Figure 2**. Within live CFSE^pos^ gated cells, the percent of PMNs, SSC-A^lo^CD172A^hi^ cells (MoCs, designated CD172A^hi^), CD14^pos^SSC-A^lo^CD172A^hi^ (designated CD14^pos^), CD16^pos^SSC-A^lo^CD172A^hi^ (designated CD16^pos^), SSC-A^lo^CD172^int^ cells (designated CD172A^int^), CD13^pos^ (cDC1 dendritic cells), NKp46^pos^ (NK cells), CD21^pos^ (B cells), CD3^pos^ (T cells), CD3^pos^CD4^pos^, CD3^pos^CD8^pos^ T cells are reported for the untreated group (UT in red) and the corticosteroid-treated (CST in black) group (5 pigs per group except for CD14^pos^ and CD16^pos^ subsets, with 3 in the UT group and 4 in the CST group). Each pig is labeled with a unique colored symbol throughout the paper. In most cases the data distribution passed the normality test and an unpaired t-test was used to identify statistically significant differences between UT and CST values; a Mann-Whitney test was used for the CD14^pos^ and CD16^pos^ subset values. No statistically significant differences were found in any case.

We next monitored the recruited cell subsets in the lung grafts of the UT and CST groups using flow cytometry analysis (**Figure 2**). Similar percentages of recruited CFSE^pos^ cells at 6 h and 10 h were found in the extracorporeal lungs in UT and CST groups (**Supplemental Figure 7**). The dominant subset among CFSE^pos^ cells in both groups was PMNs followed by MoCs. The CD14^pos^ and CD16^pos^ subsets were similarly recruited in the UT and CST groups (**Figure 2**, **Supplemental Figure 3**). In both groups other subsets represented less than 10% of the CFSE^pos^ cells and included SSC^lo^CD172A^int^ cells (not known population), CD13^pos^ cells (cDC1 (25),), NKp46^pos^ cells (natural killer), CD21^pos^ cells (mature B cells (26)), CD4^pos^CD3^pos^ and CD8^pos^CD3^pos^ T cells, with no statistically significant differences between the UT and CST groups for all populations. The recruitment of the cells in the lung led to an overall increase (versus 0 h) in the proportion of PMNs and of the monocytic cell subsets, in both the UT and CST groups, whereas the proportion of the other subsets did not increase and tended to decrease especially in the case of the CD4^pos^CD3^pos^ and CD8^pos^CD3^pos^ T cells (**Supplemental Figure 8**). Finally, we observed that at 6 h and 10 h, the proportion of monocytic cells (CD172A^hi^, CD14^pos^, CD16^pos^) originating from the donor (CFSE^neg^) generally exceeded the ones of the perfusing pig (CFSE^pos^) in the UT and CST group, from 1 to 8.8 folds depending on cases (**Supplemental Figure 9**).

Overall CS administration in the host just before the graft perfusion does not modify the representation of the recruited cell subsets in the lung.

### 3.3 The CS treatment reduces the surface expression of MHC class II and CD80/86 molecules on recruited and donor monocytic cells in lung grafts but not on AMs

**Figure 3:**
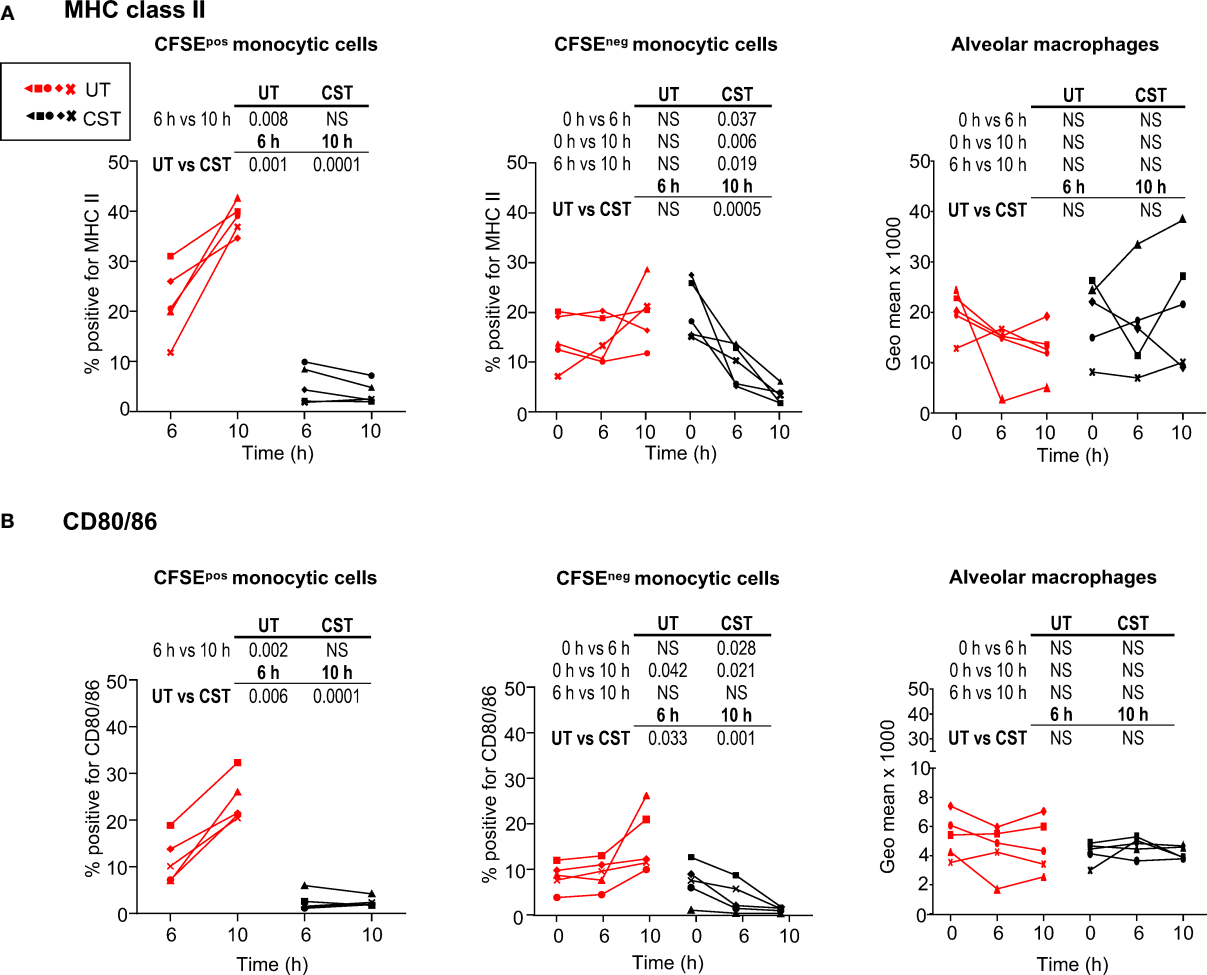
Modulation of MHC class II and CD80/86 expression on lung monocytic cells and alveolar macrophages upon cross-circulation and effects of corticosteroid treatment. **(A)** Percent of MHC class II^pos^ cells within live CFSE^pos^SSC-A^lo^CD172^hi^ cells (left panel), within live CFSE^neg^SSC-A^lo^CD172^hi^ cells (middle panel) and geometric mean expression of MHC class II on alveolar macrophages (all alveolar macrophages are MHC class II^pos^). **(B)** Same as in A for the analysis of CD80/86 expression. In **(A, B)**, 5 pigs were monitored per group. Each pig is labeled with a unique colored symbol throughout the paper (untreated group (UT) and corticosteroid-treated (CST), UT in red, CST in black). As values passed a normality test, a paired t-test was used to identify statistically significant differences between timings, and an unpaired t-test was used to identify statistically significant differences between the UT and CST groups, with p-values reported in the table above each panel. NS stands for non-significant.

Whereas the CS treatment did not modify the allogeneic cell recruitment in the graft, we assessed whether it could affect the upregulation of the MHC class II and CD80/86 found on MoCs and AMs. We found that in the UT group, the percent of MHC class II^+^ cells among the CFSE^pos^SSC^lo^CD172A^hi^ MoCs increased from 21.8 ± 7.1% at 6 h to 38.6 ± 3% at 10 h (p = 0.008), whereas in the CST group it was only 5.3 ± 3.7% at 6 h and 3.8 ± 2.2% at 10 h (p < 0.01 between UT and CST in all cases, **Figure 3A** left panel and **Supplemental Figure 10** for representative FACS raw profiles). Similarly, in the UT group, the percent of CD80/86^pos^ cells in CFSE^pos^SSC^lo^CD172A^hi^ cells increased from 11.4 ± 5% at 6 h to 24.3 ± 5% at 10 h whereas in the CST group, it was only 2.5 ± 2% at 6 h and 2.5 ± 1.0% at 10 h (p < 0.01 between UT and CST in all cases, **Figure 3B** left panel, and **Supplemental Figure 10**). We performed a similar analysis on the CFSE^neg^SSC^lo^CD172A^hi^ cells; in that case, the increase in MHC class II expression was inconsistently found in the UT group ( (19), **Figure 3A** middle panel, and **Supplemental Figure 10**). Interestingly in the CST group, the percent of MHC class II^+^ cells among the CFSE^neg^SSC^lo^CD172A^hi^ subset decreased from 20.5 ± 5.8% at 0 h to 3.4 ± 1.8% at 10 h, indicating that the donor SSC^lo^CD172A^hi^ cells were themselves responsive to the CS treatment (p = 0.0005 for the UT vs CST comparison at 10 h, **Figure 3A** middle panel). Similar findings were observed in the case of the CD80/86 expression (p = 0.001, **Figure 3B** middle panel). Finally, as AMs (SSC^hi^CD172A^hi^CD163^hi^ (19),) all express MHC class II and CD80/86, geometric mean intensities were used to show that their expression was not modified by cross-circulation nor by the CS treatment (**Figures 3A, B**, right panel).

Finally, we evaluated whether the CS treatment would increase the expression of CD163 in the context of this innate allogeneic response. Indeed CD163, a scavenger receptor for hemoglobin, is an immunomodulatory molecule that can be upregulated by CS on human monocytes *in vivo* (27). However, no clear conclusions could be obtained in our setting as reperfusion per se increased CD163 expression on pig MoCs in several instances (**Supplemental Figure 11**).

Overall these data show that CS reduces the MHC class II and CD80/86 expression on recipient and donor SSC^lo^CD172A^pos^ MoCs, whereas it does not modify their expression on AMs.

### 3.4 The corticosteroid treatment temporally modifies the inflammatory gene expression profile depending on cytokine genes and monocyte/macrophage subsets

**Figure 4:**
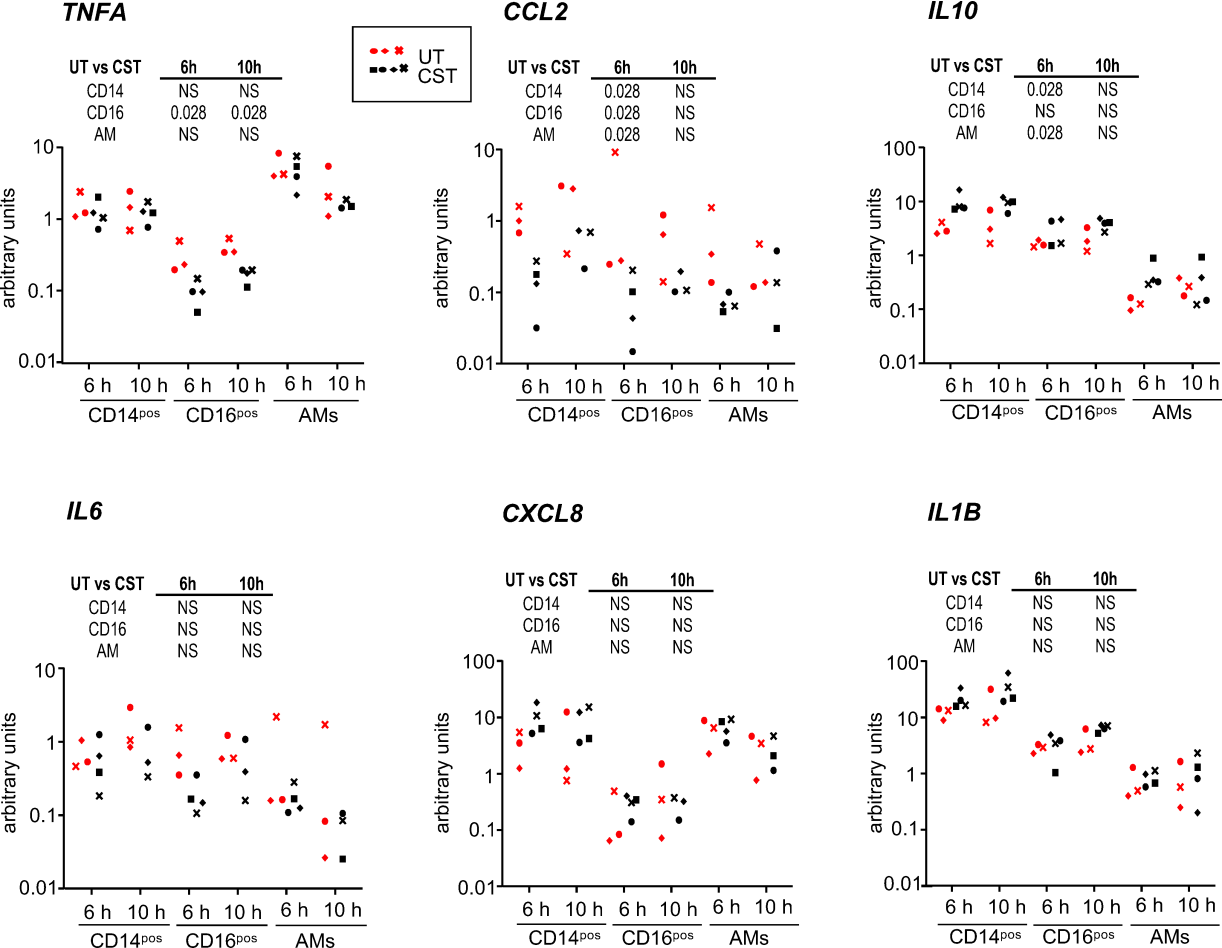
Cytokine gene expression in the CD14^pos^ and CD16^pos^ subsets and alveolar macrophages upon cross-circulation and effects of CS treatment. Gene expression arbitrary values were calculated from RT-qPCR data normalized to a house keeping gene (RSP24) and to an internal calibrator, using the 2^-ΔΔCT^ method; the gene expression data were obtained from sorted CFSE^pos^CD172A^pos^CD14^pos^ cells, CFSE^pos^CD172A^pos^CD16^pos^ cells and alveolar macrophages from the untreated group (UT) and the corticosteroid-treated group (CST) at 6 h and 10 h cross-circulation (3 pigs from UT group in red, 4 pigs from the CST group in black). See the calculation method in the material and methods. As the data did not pass a normality test, a paired Wilcoxon test was used to identify statistically significant differences between timings, and a one-tailed Mann-Whitney test was used to identify statistically significant differences between the UT and CST groups, with p-values reported in the table above each panel. NS stands for non-significant.

**Figure 5:**
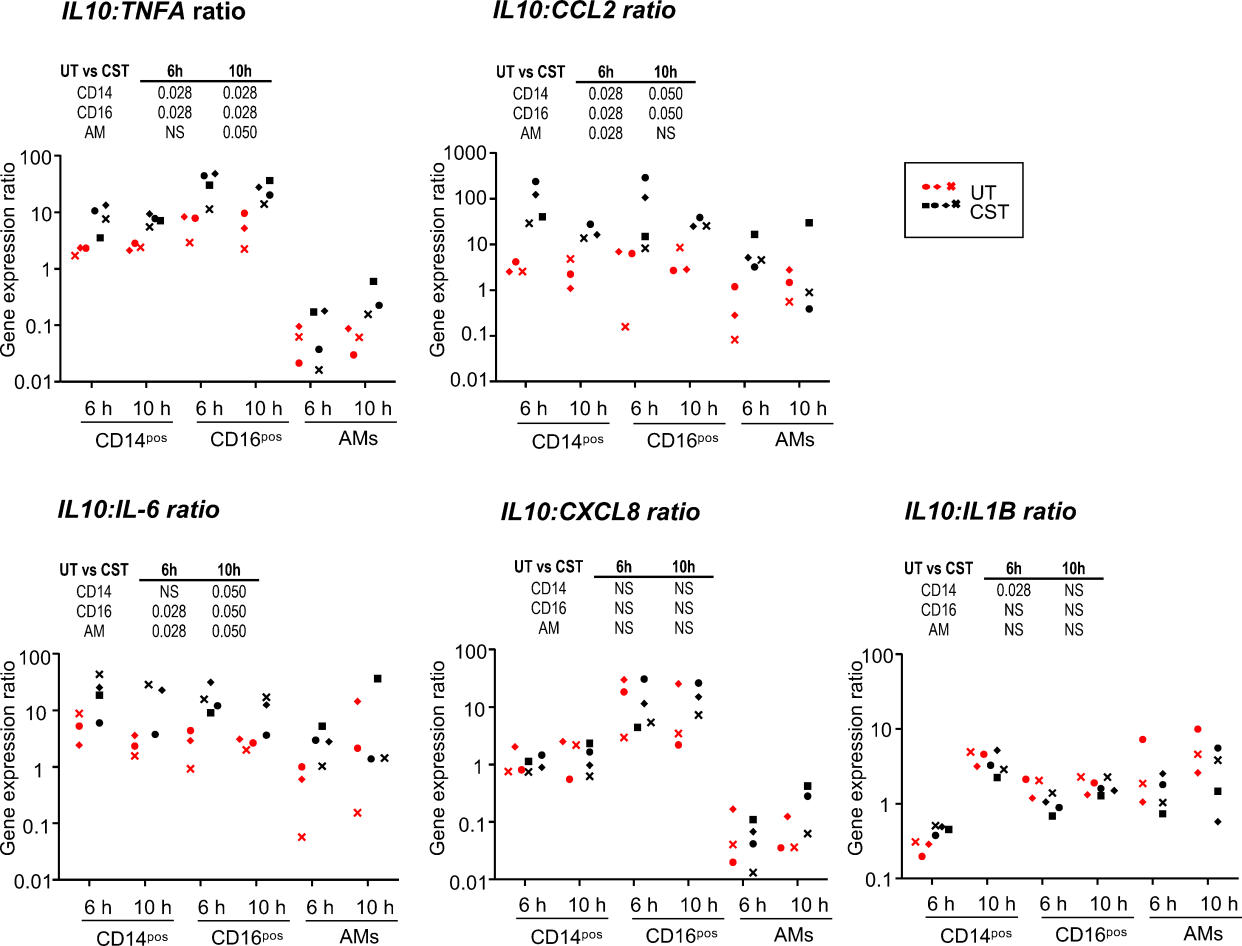
Ratio of *IL10* versus inflammatory cytokine gene expression in the CD14^pos^ and CD16^pos^ subsets and in alveolar macrophages upon cross-circulation and effects of CS treatment. A ratio between the *IL10* gene expression values and the different inflammatory cytokine values presented in **Figure 4** was calculated for sorted CFSE^pos^CD172A^pos^CD14^pos^ cells, CFSE^pos^CD172A^pos^CD16^pos^ cells, and alveolar macrophages from the untreated group (UT) and the corticosteroid-treated group (CST) at 6 h and 10 h cross-circulation (3 pigs from UT group in red, 4 pigs from the CST group in black). As the data did not pass a normality test, a paired Wilcoxon test was used to identify statistically significant differences between timings, and a one-tailed Mann-Whitney test was used to identify statistically significant differences between the UT and CST groups, with p-values reported in the table above each panel. NS stands for non-significant.

To evaluate the possible effects of the pulse of methylprednisolone on anti-inflammatory and inflammatory gene expression in monocyte/macrophage subsets, we sorted with flow cytometry the recruited CD14^pos^ and CD16^pos^ MoCs subsets as well as AMs from UT and CST groups after 6 h and 10 h cross-circulation and assessed the *TNFA, CCL2, IL6, CXCL8, IL1B* and *IL10* gene expression by RT-qPCR (**Figure 4**). We found that the *CCL2* gene expression was lower in the CST vs in the UT group for all subsets at 6 h (p = 0.028) and not at 10 h. The *TNFA* gene expression was clearly lower in the CD16^pos^ subset of the CST group both at 6 and 10 h (p = 0.028) but not in other subsets. The *IL10* gene expression was higher in the CD14^pos^ and AM subset of the CST group at 6 h (p = 0.028). No difference in expression between the UT and CST groups was obtained in any subset in the case of *IL6, CXCL8*, and *IL1B* genes. To have an estimate of the anti-inflammatory/inflammatory balance in the subsets, we calculated the *IL10*:cytokine gene expression ratios (**Figure 5**). The ratio was higher in the CST group particularly at 6 h in the case of *IL10:TNFA*, *IL10:CCL2*, and even of *IL10:IL6*, in the different subsets. The *IL10:TNFA* ratio reached very high values in the CD16^pos^ subset (33.6 ± 16.6 vs 6.4 ± 2.9 at 6 h, p = 0.028), less high in the CD14^pos^ subset (8.8 ± 4.2 vs 2.1 ± 0.4 at 6 h, p = 0.028) while this ratio remained very low (below 1 in most cases) in AMs.

Altogether the administration of CS to the perfusing pig had variable genomic effects on the cytokine gene expression and on the *IL10*:inflammatory gene expression ratios, which were generally not sustained and depended on the cytokine gene and the monocyte/macrophage cell type. The *TNFA* gene expression appeared particularly reduced in the CD16^pos^ subset, leading to high *IL10:TNFA* ratios in that subset.

## 4 Discussion

We investigated the effects of CS on the monocyte/macrophage subset responses by using a cross-circulatory platform in the pig that we recently showed to be a pertinent and robust preclinical model to study the ischemia reperfusion and innate allo-responses in lung transplantation (19). While we showed that the reperfusion selectively induced MHC class II and CD80/86 surface expression on CD16^pos^ MoCs, CS potently reduced MHC class II and CD80/86 expression on donor and recipient MoCs in the graft but not on AMs. We also found that CS did not modify the immune cell recruitment upon blood reperfusion. Finally, CS had variable effects on the cytokine gene expression depending on the cytokine genes and the cell subsets, and that were of relatively short duration (not maintained beyond 6 h in most cases). Our findings contrast with other results published previously on myeloid cell responses to CS, which can be related to multifactorial parameters including the specific signaling context of the ischemia-reperfusion in allogeneic conditions in the lung, and the monocyte/macrophage differentiation status that will be discussed next.

CS are the most widely utilized anti-inflammatory drugs. They interact with a nuclear receptor expressed in most cell types that trigger the expression of anti-inflammatory genes through direct DNA binding and downmodulates inflammatory gene expression through the trans-repression of the NF-kB and AP1 transcription factors’ action and mRNA destabilization. In addition, CS can exert non genomic actions through membrane-initiated signaling, via kinase inhibition (15, 28). The concomitant signaling pathways and preexisting open chromatin domains permitting access to the CS nuclear receptor, due to cell activation and differentiation, will condition the magnitude and direction of the gene response (16). The pulse of methylprednisolone that we administered, which represents the intraoperative use during LT in patients, may act both through non-genomic and genomic pathways (29). The non-genomic pathway may direct the profound downmodulation of membrane expression of MHC class II and CD80/86 that we observed, and genomic effects are observed on the modulation of *TNFA*, *IL10*, and *CCL2* gene expression. The effects on cytokine gene expression were also mainly visible at 6 h and less so at 10h, consistently with the methylprednisolone half-life of 1 to 3 h in human patients (30).

The effects of CS on myeloid cells are complex and not well known (15). Most studies on human myeloid cells were done with cell lines whose chromatin is strongly modified or *in vitro* on isolated monocytes and macrophage cell cultures. In such settings, CS inhibited the transcription of several pro-inflammatory cytokine genes including *IL1B, IL6, IL12, TNFA, GM-CSF, CXCL8, CCL5*, and *CCL2* (31–34), and upregulated the expression of the *IL10 gene* (35). These broad responses of cytokine genes were not observed in our context where CS did not significantly reduce the expression of the *IL6*, *IL1B*, and *CXCL8* genes but reduced the expression of *CCL2* and *TNFA*. Importantly the response to CS can strongly depend on the cell activation context: for instance in thioglycolate-induced murine macrophages, the response of the same genes was sensitive to CS in the case of TLR4 and 9 activation and resistant in the case of TLR3 activation (36). Furthermore, some agonists can induce the expression of interfering co-activators, explaining the gene-specific sensitivity or resistance to CS depending on the context (36). Therefore we can speculate that the signaling induced by the ischemia-reperfusion combined with the innate allo-recognition renders the gene response to CS variably refractory or sensitive to the CS effect. Indeed the ischemia-reperfusion during transplantation triggers multiple signaling pathways downstream of Toll-like receptor 4 (37), Dectin-1 (38), NOD-like receptor (39), and purinergic receptors (40). Furthermore, the innate allo-recognition involves recognition between CD47 and polymorphic CD172A as well as between paired immunoglobulin-like receptors (PIR) and polymorphic MHC class I whose interaction activates inflammatory cascades (9, 41). Consequently, many complex signaling pathways are likely engaged in lung transplantation that provide peculiar signaling and transcriptomic landscapes conditioning the responses to CS.

There is clear evidence that CS exerts different effects depending on the monocyte/macrophage types. These cells have different ontogenic origins, with MoCs originating from the bone marrow and AMs from embryonic progenitors and they integrate tissue-specific signals that dictate their programming and functions. In this study we found that CS effectively reduced the MHC class II and CD80/86 expression on MoCs in the lung, and not on AMs, suggesting that MoCs and AMs differentially respond to CS. In another study on human monocyte derived-DC activated by LPS, a CS (dexamethasone) did not suppress MHC class II expression, whereas it suppressed CD86 expression (42). Also, in mice, CS were found to regulate 1,035 mRNAs in monocytes and only 165 in macrophages, indicating a relative resistance to CS in differentiated macrophages (43). The differentiation stage of monocytes versus macrophages, the embryonic origin, and the tissue of residence are associated with the expression of specific signaling molecules and transcription factors that may strongly modify their receptivity to the genomic and non-genomic effects of CS.

We further refined the study of the CS effects on subsets of MoCs, as monocyte subsets were previously found to play a key role in PGD (4). In humans, an international nomenclature was adopted by the International Union of Immunological Societies, that distinguished three monocyte subsets, i.e. the classical CD14^pos^CD16^neg^, the non-classical CD14^neg^CD16^pos^ and the CD14^pos^CD16^pos^ intermediate subsets, taking into account the continuum of CD16 surface expression on human monocytes (44). At the functional level, the current view is that classical CD14^pos^CD16^neg^ monocytes are inflammatory cells rapidly recruited upon inflammation, CD14^neg^CD16^pos^ monocytes contribute to the maintenance of endothelial cell integrity, and CD14^pos^CD16^pos^ can develop into non-classical monocytes and have shown capacity for antigen presentation (45–47). The intermediate and non-classical human subsets display a high similarity in global gene expression (46). In the pig, we reported previously the identification of only two subsets, i.e. the CD14^pos^ and CD16^pos^ subsets, based on a comparative transcriptomic analysis with human and mouse monocyte subsets that did not include intermediate monocytes (8). Therefore, as others did before for human monocytes (6), we judged it pertinent to designate the pig CD16^pos^ subset as intermediate/non-classical monocytes. A complementary study using single-cell analysis would be necessary to further refine their identity in the pig. Interestingly we found that this intermediate/non-classical CD16^pos^ monocyte subset selectively upregulated antigen-presentation molecules in the context of allogeneic ischemia-reperfusion, consistent with reports in human pointing to antigen presentation abilities of the intermediate subset (45–47).

We found that CS did not decrease the recruitment of the different host immune subsets in the graft. This finding contrasts with reports documenting inhibition of inflammatory cell recruitment by CS, for instance, inhibition of the PMN recruitment induced by intratracheal lipopolysaccharide (48) and of the monocyte/macrophage recruitment induced by bleomycin in the lung (49) and carrageenan in the peritoneum (50). CS may interfere with leukocyte trafficking by modulation of adhesion molecule expression, chemokine secretion, and chemokine receptor expression. However, CS can paradoxically increase cell mobility by upregulating some chemokine receptors such as CXCR4 (51) and fMLP receptors on monocytes (52). Therefore, the effects of CS on cell migration and recruitment appear again to be cell-type- and context-dependent.

A major implication of our work is that more effective intraoperative treatments than methylprednisolone injection should be identified more strongly modulating the innate response upon lung transplantation. For instance, the pretreatment of a donor’s lungs with CS before lung procurement may improve the anti-inflammatory effect on the innate allo-response, an option that we are currently evaluating. In addition, combination with other drugs or biologicals (rapamycine, calcineurin inhibitors, GM-CSF) that is variable between transplantation centers, and the testing of other types of corticosteroids should be further explored using our cross-circulation platform for their compared efficacy versus the baseline of the current methylprednisolone treatment (3).

Our study presents limitations. First, it would have been interesting to analyze the cellular responses at time points beyond 10 h. However, later time points would require to use of the conscious pig model of cross-circulation developed by the Columbia University team (and not an anesthetized pig as used here), which implicates a challenging process of animal management (53). The conscious pig model is demanding in terms of human resources and the technical resources required to maintain blood circulation in the extracorporeal lung and would not be feasible in our logistic conditions. In addition, the CFSE labeling strategy cannot be used for over 10 hours due to the progressive extinction of the CFSE signal as documented in (19), therefore the CFSE labeling should be started at a later time point. However, while we injected CFSE into the recipient host 30 min before the onset of cross-circulation, a later injection risks potentially labeling the donor lung cells. Alternatively, as the sex of the donor’s lung differs from the perfusing pig’s one, the technique of single cell RNA-seq could be used to differentiate the donor and recipient cells, based on genes expressed by the Y chromosome and thus to study their respective transcriptomic responses at different time points. Another limitation of our study is that a complete transplantation might modify the results obtained with cross-circulation. Indeed compared to cross-circulation, full transplantation is expected to generate a higher degree of local and systemic inflammation, related to the surgical stress induced by cuts, cauterization, and hemodynamic perturbations. Nevertheless, cross-circulation and full transplantation share similar signaling pathways related to ischemia-reperfusion and allogenicity in the lung, making cross-circulation a pertinent surrogate platform of investigation. The cross-circulation platform offers experimental simplicity and controllability and it leads to a high degree of experimental reproducibility that may not have been reached with full transplantation. Finally, cross-circulation is far more acceptable from an ethical point of view, an important parameter in the management of animal experiments.

Altogether this work shows the partial effect of the intraoperative treatment with CS on the innate allogeneic response and emphasizes the CS specific effects depending on myeloid subsets. It also has important implications for designing more effective anti-inflammatory and immunoregulatory therapies to be applied during the surgery, to optimally control response to ischemia-reperfusion and allogenic innate responses encountered in the transplantation process, and therefore lead to a reduction in the burden of the subsequent immunosuppressive treatments and improving the outcome of lung transplanted patients.

## Data availability statement

The original contributions presented in the study are included in the article/**Supplementary Material**. Further inquiries can be directed to the corresponding author.

## Ethics statement

The experiments were approved by the COMETHEA ethic committee under the APAFIS number authorization 25174-2020011414322379 and were authorized by the French “ministère de l’enseignement supérieur et de la recherche”.

## Author contributions

MG: Conceptualization, Formal Analysis, Investigation, Methodology, Writing – review & editing. FP: Formal Analysis, Investigation, Methodology, Writing – review & editing, Data curation, Visualization. MH: Formal Analysis, Investigation, Methodology, Visualization, Writing – review & editing. JE: Formal Analysis, Investigation, Methodology, Writing – review & editing. CG: Investigation, Methodology, Writing – review & editing. CU: Investigation, Methodology, Writing – review & editing. MB: Investigation, Methodology, Writing – review & editing. GE: Writing – review & editing, Resources. CR: Methodology, Writing – review & editing, Investigation. VG: Methodology, Writing – review & editing, Investigation. JW: Investigation, Methodology, Writing – review & editing. MLG: Investigation, Methodology, Writing – review & editing. AM: Investigation, Writing – review & editing. AR: Investigation, Writing – review & editing. PD: Investigation, Writing – review & editing. IS-C: Conceptualization, Funding acquisition, Investigation, Methodology, Resources, Supervision, Data curation, Formal Analysis, Validation, Visualization, Writing – original draft. ES: Methodology, Writing – review & editing, Conceptualization, Funding acquisition, Investigation, Project administration, Resources, Supervision.

## References

[1] DerHovanessian A Wallace WD Lynch JP 3rd Belperio JA Weigt SS. Chronic lung allograft dysfunction: evolving concepts and therapies. Semin Respir Crit Care Med (2018) 39(2):155–71. doi: 10.1055/s-0037-1618567 29579768

[2] Ochando J Braza MS. Nanoparticle-based modulation and monitoring of antigen-presenting cells in organ transplantation. Front Immunol (2017) 8:1888. doi: 10.3389/fimmu.2017.01888 29312352 PMC5743935

[3] Colombo M Marongiu L Mingozzi F Marzi R Cigni C Facchini FA. Specific immunosuppressive role of nanodrugs targeting calcineurin in innate myeloid cells. iScience (2022) 25(10):105042. doi: 10.1016/j.isci.2022.105042 36124235 PMC9482116

[4] Kurihara C Lecuona E Wu Q Yang W Nunez-Santana FL Akbarpour M. Crosstalk between non-classical monocytes and alveolar macrophages mediates transplant ischemia-reperfusion injury through classical monocyte recruitment. JCI Insight (2021) 6(6):e147282. doi: 10.1172/jci.insight.147282 33621212 PMC8026186

[5] Scott CL Henri S Guilliams M. Mononuclear phagocytes of the intestine, the skin, and the lung. Immunol Rev (2014) 262(1):9–24. doi: 10.1111/imr.12220 25319324

[6] Mulder K Patel AA Kong WT Piot C Halitzki E Dunsmore G. Cross-tissue single-cell landscape of human monocytes and macrophages in health and disease. Immunity (2021) 54(8):1883–1900.e5. doi: 10.1016/j.immuni.2021.07.007 34331874

[7] Teh YC Ding JL Ng LG Chong SZ. Capturing the fantastic voyage of monocytes through time and space. Front Immunol (2019) 10:834. doi: 10.3389/fimmu.2019.00834 31040854 PMC6476989

[8] Vu Manh TP Elhmouzi-Younes J Urien C Ruscanu S Jouneau L Bourge M. Defining Mononuclear Phagocyte Subset Homology across Several Distant Warm-Blooded Vertebrates through Comparative Transcriptomics. Front Immunol (2015) 6:299. doi: 10.3389/fimmu.2015.00299 26150816 PMC4473062

[9] Zhao D Abou-Daya KI Dai H Oberbarnscheidt MH Li XC Lakkis FG. Innate allorecognition and memory in transplantation. Front Immunol (2020) 11:918. doi: 10.3389/fimmu.2020.00918 32547540 PMC7270276

[10] Ochando J Ordikhani F Boros P Jordan S. The innate immune response to allotransplants: mechanisms and therapeutic potentials. Cell Mol Immunol (2019) 16(4):350–6. doi: 10.1038/s41423-019-0216-2 PMC6462017 30804476

[11] Porteous MK Lee JC. Primary graft dysfunction after lung transplantation. Clin Chest Med (2017) 38(4):641–54. doi: 10.1016/j.ccm.2017.07.005 29128015

[12] DerHovanessian A Weigt SS Palchevskiy V Shino MY Sayah DM Gregson AL. The role of tgf-beta in the association between primary graft dysfunction and bronchiolitis obliterans syndrome. Am J Transplant (2016) 16(2):640–9. doi: 10.1111/ajt.13475 PMC4946573 26461171

[13] Scheffert JL Raza K. Immunosuppression in lung transplantation. J Thorac Dis (2014) 6(8):1039–53. doi: 10.3978/j.issn.2072-1439.2014.04.23 PMC4133546 25132971

[14] Brann SH Geier SS Timofeeva O Shigemura N Cordova F Toyoda Y. Perioperative Care for Lung Transplant Recipients: A Multidisciplinary Approach. In: Vitin A, editor. Perioperative Care for Organ Transplant Recipient. Rijeka: IintechOpen (2019).

[15] Ehrchen JM Roth J Barczyk-Kahlert K. More than suppression: glucocorticoid action on monocytes and macrophages. Front Immunol (2019) 10:2028. doi: 10.3389/fimmu.2019.02028 31507614 PMC6718555

[16] Franco LM Gadkari M Howe KN Sun J Kardava L Kumar P. Immune regulation by glucocorticoids can be linked to cell type-dependent transcriptional responses. J Exp Med (2019) 216(2):384–406. doi: 10.1084/jem.20180595 30674564 PMC6363437

[17] Wang A Ali A Keshavjee S Liu M Cypel M. Ex vivo lung perfusion for donor lung assessment and repair: A review of translational interspecies models. Am J Physiol Lung Cell Mol Physiol (2020) 319(6):L932–40. doi: 10.1152/ajplung.00295.2020 32996780

[18] Sachs DH. Tolerance: of mice and men. J Clin Invest (2003) 111(12):1819–21. doi: 10.1172/JCI18926 PMC161433 12813017

[19] Glorion M Pascale F Estephan J Huriet M Gouin C Urien C. A cross-circulatory platform for monitoring innate allo-responses in lung grafts. PloS One (2023) 18(5):e0285724. doi: 10.1371/journal.pone.0285724 37253049 PMC10228766

[20] Roederer M. Compensation in flow cytometry. Curr Protoc Cytom (2002) Chapter 1:Unit 1:14. doi: 10.1002/0471142956.cy0114s22 18770762

[21] Moreno S Alvarez B Poderoso T Revilla C Ezquerra A Alonso F. Porcine monocyte subsets differ in the expression of ccr2 and in their responsiveness to ccl2. Vet Res (2010) 41(5):76. doi: 10.1051/vetres/2010048 20670605 PMC2941139

[22] Mariscal A Caldarone L Tikkanen J Nakajima D Chen M Yeung J. Pig lung transplant survival model. Nat Protoc (2018) 13(8):1814–28. doi: 10.1038/s41596-018-0019-4 30072720

[23] O'Neill JD Guenthart BA Kim J Chicotka S Queen D Fung K. Circulation for extracorporeal support and recovery of the lung. Nat Biomed eng (2017) 1(1):1. doi: 10.1038/s41551-017-0037

[24] Kelly Wu W Guenthart BA O'Neill JD Hozain AE Tipograf Y Ukita R. Technique for xenogeneic cross-circulation to support human donor lungs ex vivo. J Heart Lung Transplant (2022) 42(3):335–344. doi: 10.1016/j.healun.2022.11.002 PMC9985920 36456408

[25] Bernelin-Cottet C Urien C McCaffrey J Collins D Donadei A McDaid D. Electroporation of a nanoparticle-associated DNA vaccine induces higher inflammation and immunity compared to its delivery with microneedle patches in pigs. J Control Release (2019) 308:14–28. doi: 10.1016/j.jconrel.2019.06.041 31265882

[26] Sinkora M Stepanova K Sinkorova J. Different anti-cd21 antibodies can be used to discriminate developmentally and functionally different subsets of B lymphocytes in circulation of pigs. Dev Comp Immunol (2013) 39(4):409–18. doi: 10.1016/j.dci.2012.10.010 23178404

[27] Krejsek J Mandak J Kunes P Lonsky V Kolackova M Jankovicova K. Impact of methylprednisolone in priming solution of cardiopulmonary bypass on anti-inflammatory cd163 receptor during cardiac surgery. Perfusion (2012) 27(4):284–91. doi: 10.1177/0267659112439595 22354894

[28] Panettieri RA Schaafsma D Amrani Y Koziol-White C Ostrom R Tliba O. Non-genomic effects of glucocorticoids: an updated view. Trends Pharmacol Sci (2019) 40(1):38–49. doi: 10.1016/j.tips.2018.11.002 30497693 PMC7106476

[29] Corral-Gudino L Cusacovich I Martin-Gonzalez JI Muela-Molinero A Abadia-Otero J Gonzalez-Fuentes R. Effect of intravenous pulses of methylprednisolone 250 mg versus dexamethasone 6 mg in hospitalised adults with severe covid-19 pneumonia: an open-label randomised trial. Eur J Clin Invest (2023) 53(1):e13881. doi: 10.1111/eci.13881 36169086 PMC9538428

[30] Leussink VI Jung S Merschdorf U Toyka KV Gold R. High-dose methylprednisolone therapy in multiple sclerosis induces apoptosis in peripheral blood leukocytes. Arch Neurol (2001) 58(1):91–7. doi: 10.1001/archneur.58.1.91 11176941

[31] Cain DW Cidlowski JA. Immune regulation by glucocorticoids. Nat Rev Immunol (2017) 17(4):233–47. doi: 10.1038/nri.2017.1 PMC9761406 28192415

[32] Larsson S Linden M. Effects of a corticosteroid, budesonide, on production of bioactive il-12 by human monocytes. Cytokine (1998) 10(10):786–9. doi: 10.1006/cyto.1998.0362 9811532

[33] Ma W Gee K Lim W Chambers K Angel JB Kozlowski M. Dexamethasone inhibits il-12p40 production in lipopolysaccharide-stimulated human monocytic cells by down-regulating the activity of C-jun N-terminal kinase, the activation protein-1, and nf-kappa B transcription factors. J Immunol (2004) 172(1):318–30. doi: 10.4049/jimmunol.172.1.318 14688340

[34] Escoter-Torres L Caratti G Mechtidou A Tuckermann J Uhlenhaut NH Vettorazzi S. Fighting the fire: mechanisms of inflammatory gene regulation by the glucocorticoid receptor. Front Immunol (2019) 10:1859. doi: 10.3389/fimmu.2019.01859 31440248 PMC6693390

[35] King EM Holden NS Gong W Rider CF Newton R. Inhibition of nf-kappab-dependent transcription by mkp-1: transcriptional repression by glucocorticoids occurring via P38 mapk. J Biol Chem (2009) 284(39):26803–15. doi: 10.1074/jbc.M109.028381 PMC2785369 19648110

[36] Ogawa S Lozach J Benner C Pascual G Tangirala RK Westin S. Molecular determinants of crosstalk between nuclear receptors and toll-like receptors. Cell (2005) 122(5):707–21. doi: 10.1016/j.cell.2005.06.029 PMC1430687 16143103

[37] Zanotti G Casiraghi M Abano JB Tatreau JR Sevala M Berlin H. Novel critical role of toll-like receptor 4 in lung ischemia-reperfusion injury and edema. Am J Physiol Lung Cell Mol Physiol (2009) 297(1):L52-63. doi: 10.1152/ajplung.90406.2008 PMC2711808 19376887

[38] Braza MS van Leent MMT Lameijer M Sanchez-Gaytan BL Arts RJW Perez-Medina C. Inhibiting inflammation with myeloid cell-specific nanobiologics promotes organ transplant acceptance. Immunity (2018) 49(5):819–28 e6. doi: 10.1016/j.immuni.2018.09.008 30413362 PMC6251711

[39] Ghafouri-Fard S Shoorei H Poornajaf Y Hussen BM Hajiesmaeili Y Abak A. Nlrp3: role in ischemia/reperfusion injuries. Front Immunol (2022) 13:926895. doi: 10.3389/fimmu.2022.926895 36238294 PMC9552576

[40] Haywood N Ta HQ Rotar E Daneva Z Sonkusare SK Laubach VE. Role of the purinergic signaling network in lung ischemia-reperfusion injury. Curr Opin Organ Transplant (2021) 26(2):250–7. doi: 10.1097/MOT.0000000000000854 PMC9270688 33651003

[41] Lakkis FG Li XC. Innate allorecognition by monocytic cells and its role in graft rejection. Am J Transplant (2018) 18(2):289–92. doi: 10.1111/ajt.14436 PMC5775052 28722285

[42] Manome H Aiba S Singh S Yoshino Y Tagami H. Dexamethasone and cyclosporin a affect the maturation of monocyte-derived dendritic cells differently. Int Arch Allergy Immunol (2000) 122(1):76–84. doi: 10.1159/000024361 10859472

[43] Wang C Nanni L Novakovic B Megchelenbrink W Kuznetsova T Stunnenberg HG. Extensive epigenomic integration of the glucocorticoid response in primary human monocytes and in vitro derived macrophages. Sci Rep (2019) 9(1):2772. doi: 10.1038/s41598-019-39395-9 30809020 PMC6391480

[44] Ziegler-Heitbrock L Ancuta P Crowe S Dalod M Grau V Hart DN. Nomenclature of monocytes and dendritic cells in blood. Blood (2010) 116(16):e74–80. doi: 10.1182/blood-2010-02-258558 20628149

[45] Guilliams M Mildner A Yona S. Developmental and functional heterogeneity of monocytes. Immunity (2018) 49(4):595–613. doi: 10.1016/j.immuni.2018.10.005 30332628

[46] Wong KL Tai JJ Wong WC Han H Sem X Yeap WH. Gene expression profiling reveals the defining features of the classical, intermediate, and nonclassical human monocyte subsets. Blood (2011) 118(5):e16–31. doi: 10.1182/blood-2010-12-326355 21653326

[47] Jakubzick CV Randolph GJ Henson PM. Monocyte differentiation and antigen-presenting functions. Nat Rev Immunol (2017) 17(6):349–62. doi: 10.1038/nri.2017.28 28436425

[48] O'Leary EC Marder P Zuckerman SH. Glucocorticoid effects in an endotoxin-induced rat pulmonary inflammation model: differential effects on neutrophil influx, integrin expression, and inflammatory mediators. Am J Respir Cell Mol Biol (1996) 15(1):97–106. doi: 10.1165/ajrcmb.15.1.8679228 8679228

[49] Khalil N Whitman C Zuo L Danielpour D Greenberg A. Regulation of alveolar macrophage transforming growth factor-beta secretion by corticosteroids in bleomycin-induced pulmonary inflammation in the rat. J Clin Invest (1993) 92(4):1812–8. doi: 10.1172/JCI116771 PMC288344 7691887

[50] Lee SB Lee HW Singh TD Li Y Kim SK Cho SJ. Visualization of macrophage recruitment to inflammation lesions using highly sensitive and stable radionuclide-embedded gold nanoparticles as a nuclear bio-imaging platform. Theranostics (2017) 7(4):926–34. doi: 10.7150/thno.17131 PMC5381254 28382164

[51] Caulfield J Fernandez M Snetkov V Lee T Hawrylowicz C. Cxcr4 expression on monocytes is up-regulated by dexamethasone and is modulated by autologous cd3+ T cells. Immunology (2002) 105(2):155–62. doi: 10.1046/j.0019-2805.2001.01359.x PMC1782655 11872090

[52] Pieters WR Houben LA Koenderman L Raaijmakers JA. C5a-induced migration of human monocytes is primed by dexamethasone. Am J Respir Cell Mol Biol (1995) 12(6):691–6. doi: 10.1165/ajrcmb.12.6.7766432 7766432

[53] Hozain AE Tipograf Y Pinezich MR Cunningham KM Donocoff R Queen D. Multiday maintenance of extracorporeal lungs using cross-circulation with conscious swine. J Thorac Cardiovasc Surg (2019) 159(4):1640–53 e18. doi: 10.1016/j.jtcvs.2019.09.121 31761338 PMC7094131

