# Supplementary material for: Differential early response of monocyte/macrophage subsets to intra-operative corticosteroid administration in lung transplantation
Source: Front Immunol. 2023 Oct 24;14:1281546. doi: 10.3389/fimmu.2023.1281546 (PMC10628533; doi:10.3389/fimmu.2023.1281546)
Supplement: Supplementary file 1 [file DataSheet_1.pdf]

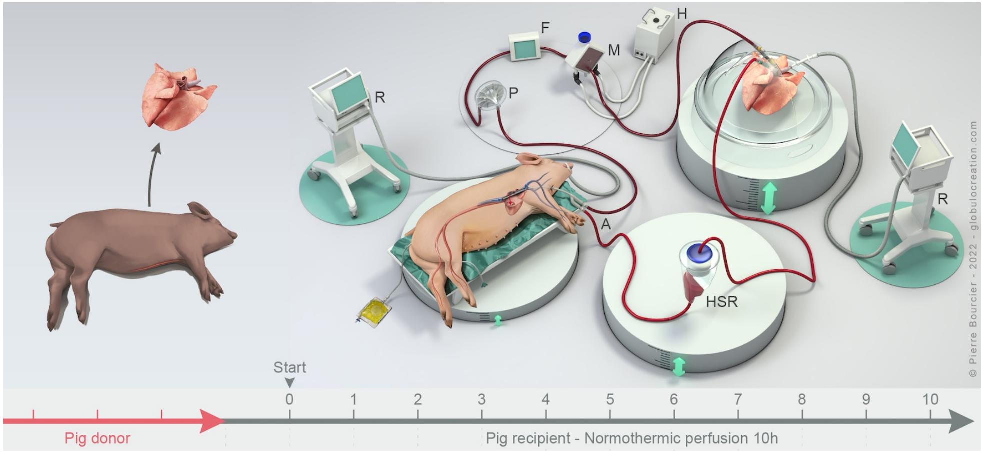

Supplemental Figure 1. Experimental scheme extracted from the supplementary material of our recent paper [Glorion, 2023, PMID 37253049]. Experimental overview of the cross-circulation platform coupled to cell mapping. A double lumen canula, HSR hard-shell reservoir, R ventilator, H heater, M membrane, F flow-meter, P pump. CFSE (25 mg) was injected 30 min before cross-circulation whereas methylprednisolone (CS, 20 mg/kg) was injected 60 min before cross-circulation in the CST group only. Blood and lung tissue biopsies were collected at different time points. Blood was used for biochemical profiling in plasma and for blood cell counts. Lung tissue was used for enzymatic dissociation ($CFSE^{pos}$ and $CFSE^{neg}$ cells composition in the tissue), and for formalin-fixed paraffin-embedded section generation (histological analyses).

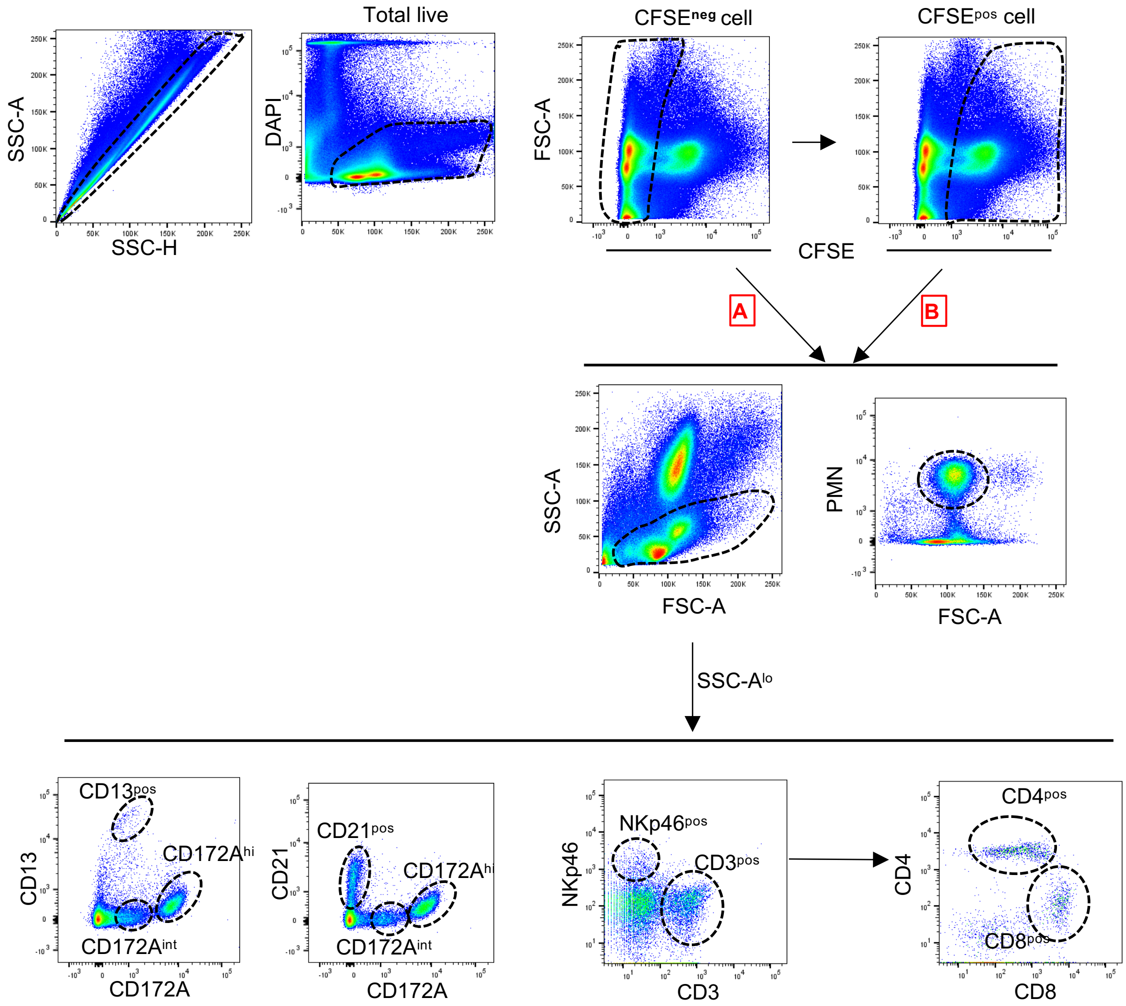

Supplemental Figure 2, extracted from the supplementary material of our recent paper [Glorion, 2023, PMID 37253049]. Gating strategy for the pig lung immune cell analyses. After gating on singlet and live cells, the workflow A was followed for CFSE^{neg} cells analyses and the workflow B was followed for CFSE^{pos} cell analyses. Except for PMNs, cells were analyzed using an intermediate gate on SSC-A^{lo} cells to avoid noises from PMNs. PMNs, CD172A^{hi} (monocytic cells, MoCs), CD172A^{int}, CD13^{pos} (cDC1 dendritic cell subset), NKp46^{pos} (NK-cells), CD21^{pos}(B-cells), CD3^{pos} T-cells, CD3^{pos}CD4^{pos} T-cells, CD3^{pos}CD8^{pos} T-cell subsets are shown. The gating strategy for the CD14^{pos} and CD16^{pos} cells is illustrated in Fig. 1.

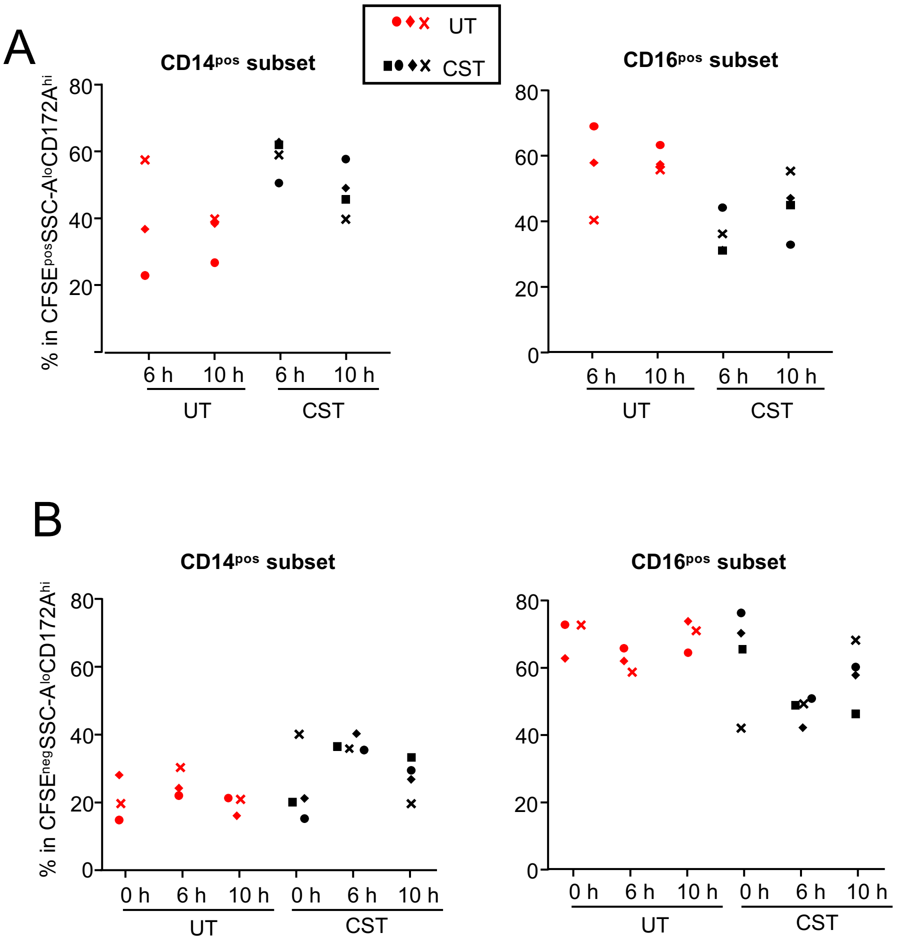

Supplemental Figure 3. Proportion of the CD14^{pos} and CD16^{pos} monocytic subsets within the live SSC-A^{lo}CD172A^{hi} subset in the UT and CST groups. A. The CD14^{pos} and CD16^{pos} subsets were selected in the CFSE^{pos}SSC-A^{lo}CD172A^{hi} cells as shown in Figure 1 for the UT (3 pigs) and CST group (4 pigs). The percent CD14^{pos} and CD16^{pos} cells in live CFSE^{pos}CCS-A^{lo}CD172A^{hi} for each pig is represented with the same symbols throughout the paper, UT in red, CST in black. **B.** The CD14^{pos} and CD16^{pos} subsets were selected in the CFSE^{neg}SSC-A^{lo}CD172A^{hi} cells as shown in Supplemental Figure 4 for the UT (3 pigs) and CST group (4 pigs). As the data did not follow a normal distribution, a paired Wilcoxon test was performed to compare values across timing and a 2-tailed Mann-Whitney test was performed to compare values across groups (UT vs CST). No statistically significant differences were found.

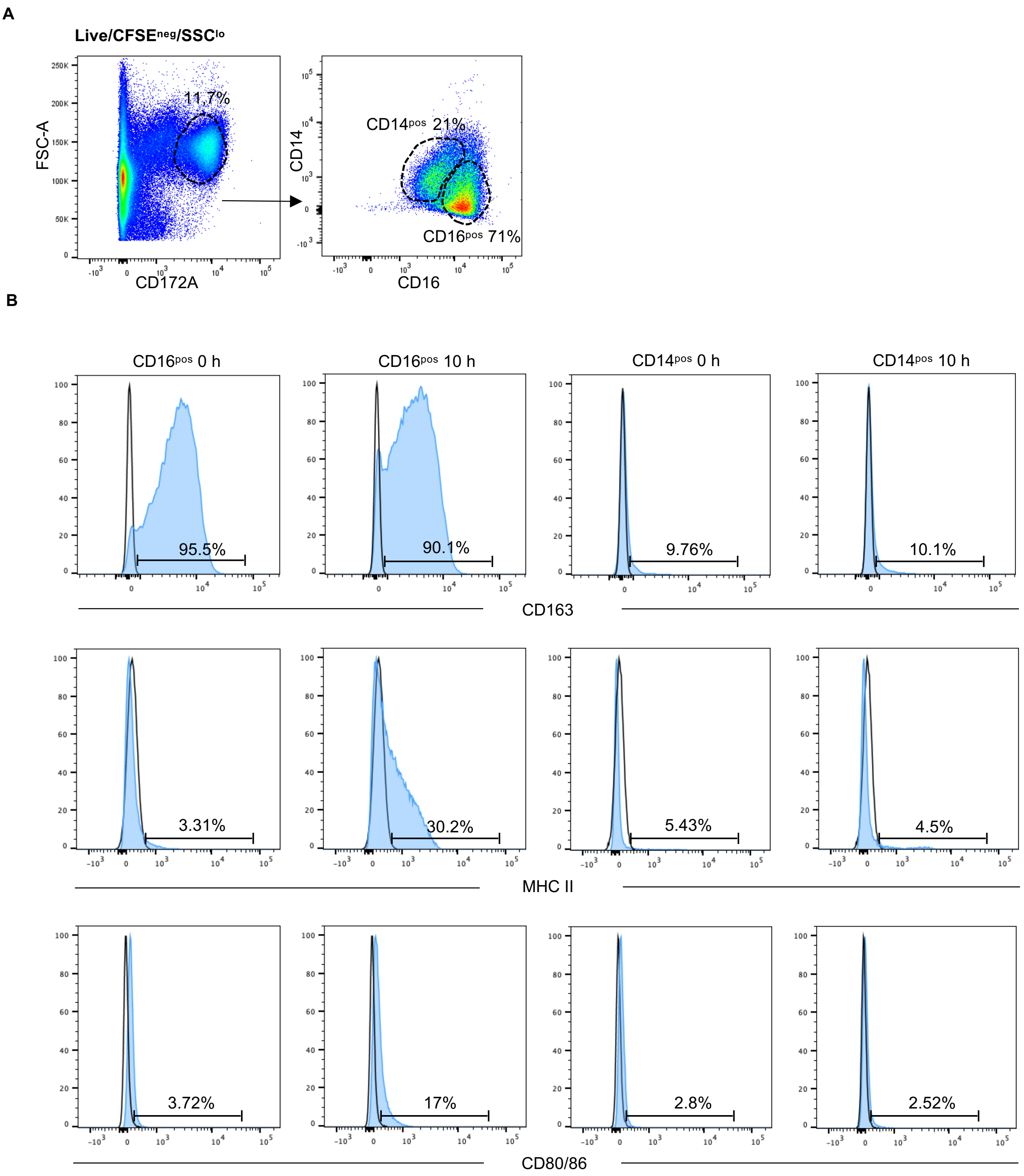

Supplemental Figure 4. The CFSE^{neg} monocytic cells in lung grafts include CD14^{pos} and CD16^{pos} subsets before and after reperfusion. A. The live CFSE^{neg}SSC-A^{lo} cells from a representative cross-circulation at 10 h were gated on the CD172A^{hi} cells that were further split in CD14^{pos} and CD16^{pos} cells. This representative experiment is depicted as a cross symbol in the different figures. **B.** The expression of CD163, MHC class II and CD80/86 on the CD14^{pos} and CD16^{pos} cells among the live CFSE^{neg}SSC-A^{lo}CD172A^{hi} lung cells is depicted (filled blue histogram) versus an IgG2a isotype control (black line) at 0 h (= donor at procurement) and 10 h post-reperfusion. The percent positive cells is indicated.

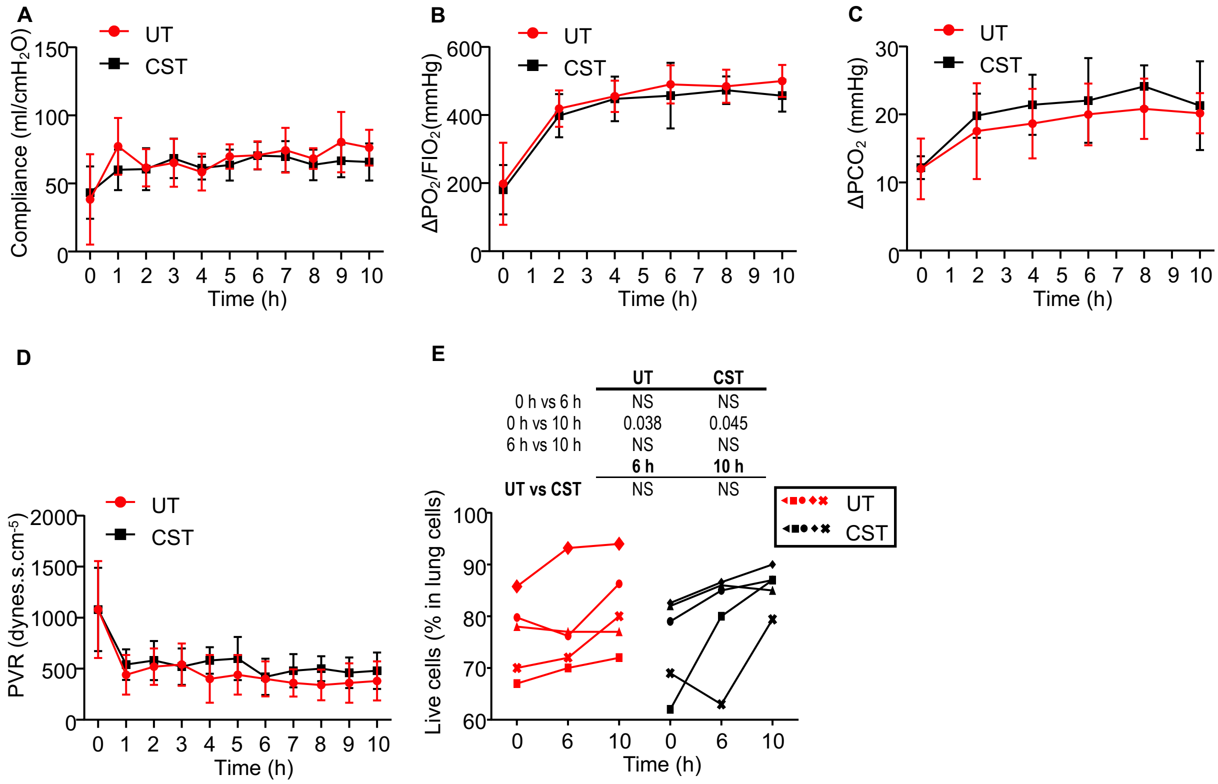

Supplemental Figure 5. Extracorporeal lung stability and performance throughout 10 h of cross-circulation. A. Static compliance. **B.** $\Delta PO_2/FIO_2$: venous PO_2 – arterial PO_2/ FIO_2. **C.** ΔPCO_2 = arterial PCO_2 – venous PCO_2 **. D.** Pulmonary Vascular Resistance (PVR): (pulmonary arterial pressure - left atrium pressure) x 80/flow rate. **E.** Live cells in total lung cells were evaluated with exclusion of DAPI staining from enzymatically dissociated lung fragments collected at 0, 6, 10 h. **In A, B, C, D** : all values represent mean ± standard deviation, untreated group (UT) values in red (n = 5) and corticosteroid-treated (CST) values (n = 4) in black. The values in the 2 groups were compared by a two-tailed Mann-Whitney test at each timing and no significant differences were obtained. **In E**, as the data distribution passed the normality test, a paired t-test test was used to identify statistically significant differences between timings and an unpaired t-test was used to identify statistically significant differences between the UT and CST groups, with p-values reported in a table above the panel.

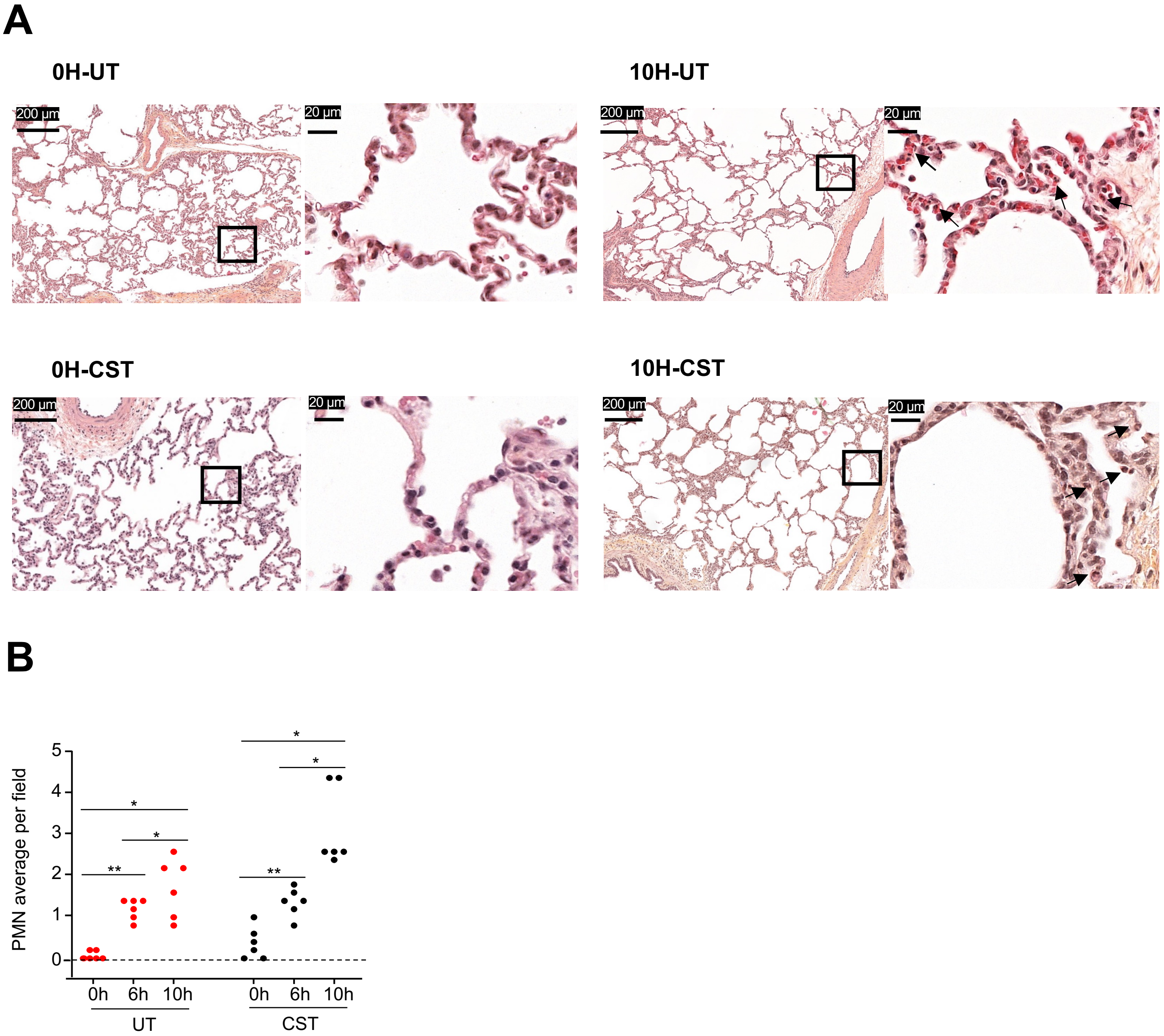

Supplemental Figure 6. Polymorphonuclear cell infiltration upon cross-circulation. A. Histological sections of the lungs before (0 h) and 10 h after cross-circulation in one representative example of the UT and CST group. **B.** The mean number of PMNs per high power field ($7x10^4$ µm^2 area) was calculated from five randomly-selected fields per slide, and 3 slides per sample were observed from two pigs per group at each timing. As the data distribution did not pass the normality test, a two-tailed Mann-Whitney test was performed between timing.

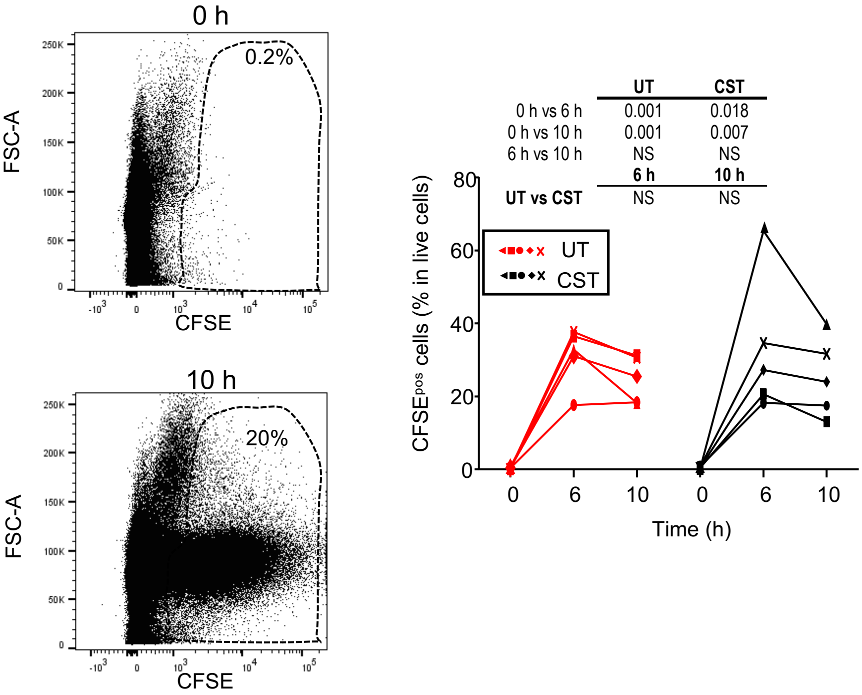

Supplemental Figure 7. Detection of CFSE^{pos} cells in the lung upon cross-circulation. Lung biopsies were collected at 0 h, 6 h and 10 h of cross-circulation and a single cell suspension was generated by enzymatic treatment. Left, the FACS profiles at 0 and 10 h of one representative experiment are shown and the % CFSE^{pos} among DAPI^{neg} live cells is indicated. Right, the proportion of CFSE^{pos} cells among live lung cells from the untreated group (UT) and corticosteroid-treated (CST) group is shown (5 pigs/group). Each pig is labelled with a unique colored symbol throughout the paper. As the data distribution passed the normality test, a paired t-test test was used to identify statistically significant differences between timings and an unpaired t-test was used to identify statistically significant differences between the UT and CST groups, with p-values reported in a table above the panel.

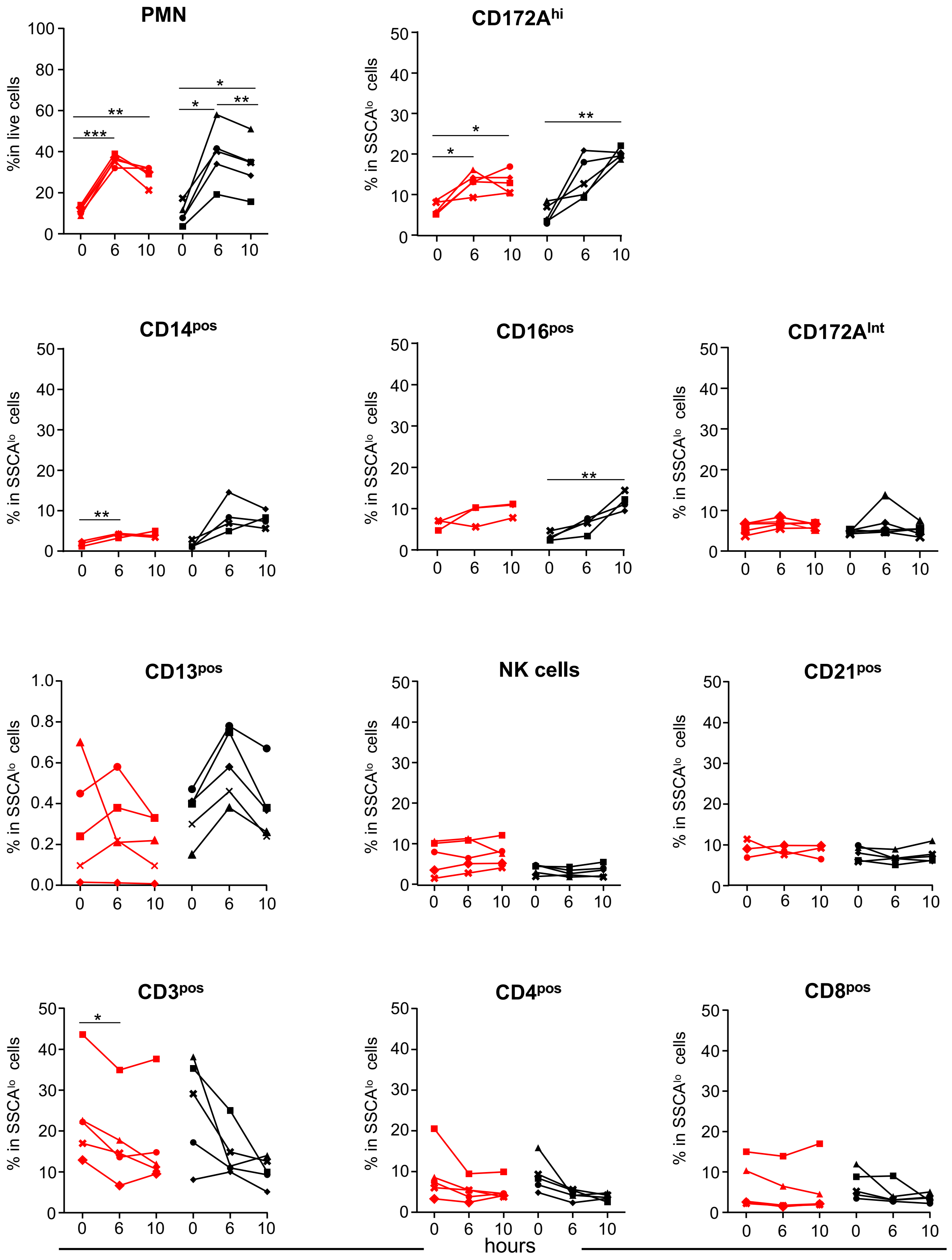

Supplemental Figure 8. Representation of the different immune cell subsets within the total lung cells at 0, 6 and 10 h cross-circulation. Cells were gated as presented in the workflow for total lung cell analysis in Supplemental Figure 2. For the PMNs, the percent cells is shown versus the total live cells (5 pigs/group). For the other subsets, the percent of the subset among total live SSC-A^{lo} cells is reported (5 pigs /groups except 3 pigs (UT) and 4 pigs (CST) for the CD14^{pos} and CD16^{pos} cells). Each pig is labelled with a unique colored symbol throughout the paper (UT in red, CST in black). When the data followed a normal distribution, a paired t-test were performed to compare the data between timing, alternatively a paired Wilcoxon test was performed.

A. 6 h cross-circulation

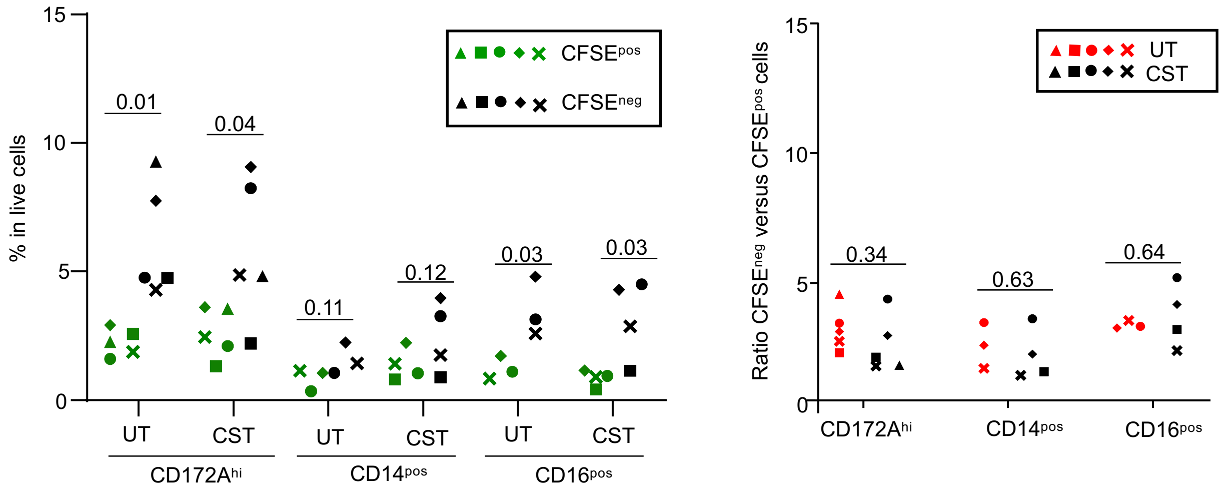

B. 10 h cross-circulation

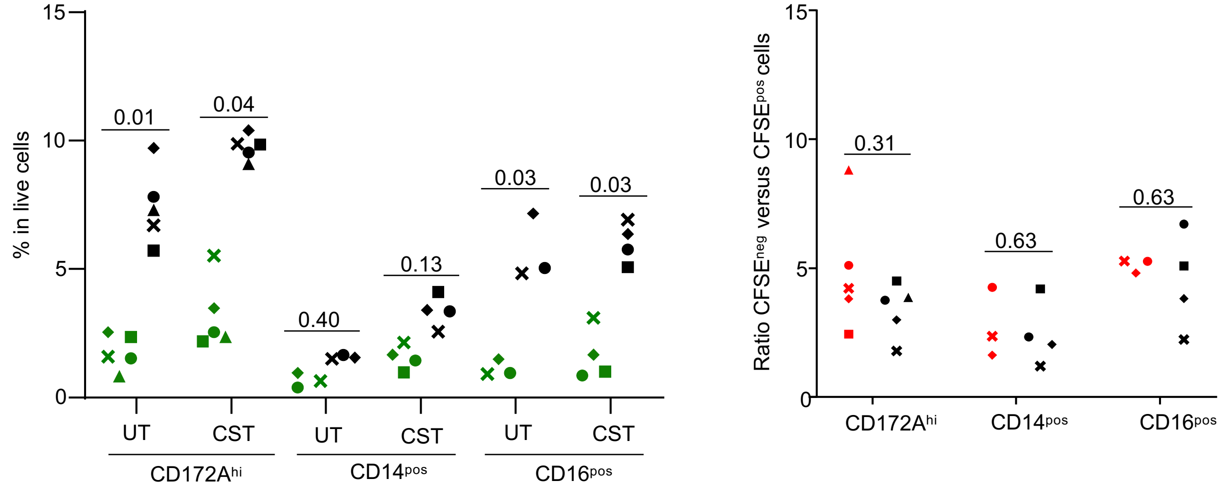

Supplemental Figure 9: Comparisons of the frequencies of CFSE^{pos} (recipient) and CFSE^{neg} (mainly donor) cells for each monocytic cell subset. A. Comparisons at 6 h cross-circulation. Left panel: percentages in live cells of the CFSE^{pos} and CFSE^{neg} monocytic cell subsets, in the UT and CST groups. Right panel: ratios of CFSE^{neg} versus CFSE^{pos} cells for each monocytic cell subsets, in the UT and CST groups. When the data followed a normal distribution, a paired t-test (comparison between CFSE^{pos} and CFSE^{neg}) and a unpaired t-test (comparison between groups) were performed to compare the data, alternatively a Wilcoxon test was performed. **B.** Comparisons at 10 h cross-circulation, following the same path as in A. Note that as mentionned in main text, 5 pigs were groups were used for the CD172A^{pos} cells, and 3 pigs (UT) and 4 pigs (CST) for the CD14^{pos} and CD16^{pos} cells.

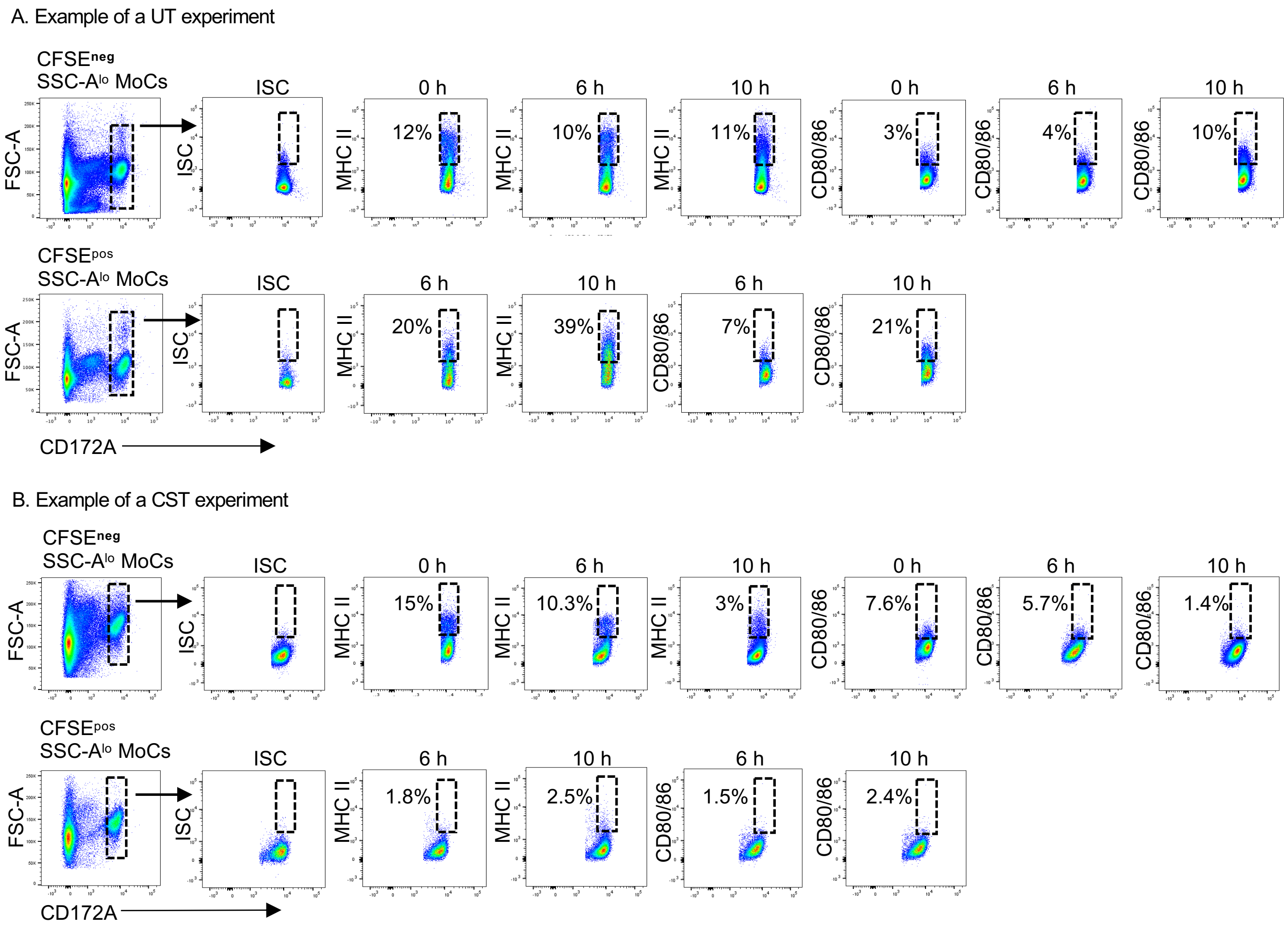

Supplemental Figure 10. MHC class II and CD80/86 expression analysis on the CFSE^{neg}SSC-A^{lo}CD172A^{hi} cells and CFSE^{pos}SSC-A^{lo}CD172A^{hi} from representative UT and CST pigs. Pig lung cells from cross-circulation experiments with a UT **(A)** and a CST **(B)** pig were analyzed for MHC class II and CD80/CD86 expression on live CFSE^{neg}SSC-A^{lo}CD172A^{hi} cells and CFSE^{pos}SSC-A^{lo}CD172A^{hi} cells at the indicated timing (0, 6, 10 h). An IgG2a isotype control (ISC) was done on a pool of lung cells from the 0, 6 and 10 h biopsies (see material and methods). The percent positive cells among CD172A^{hi} cells is depicted. The “Example of a UT experiment” is the animal represented by a red filled circle in all figures. The “Example of a CST experiment” is the animal represented by a black cross in all figures.

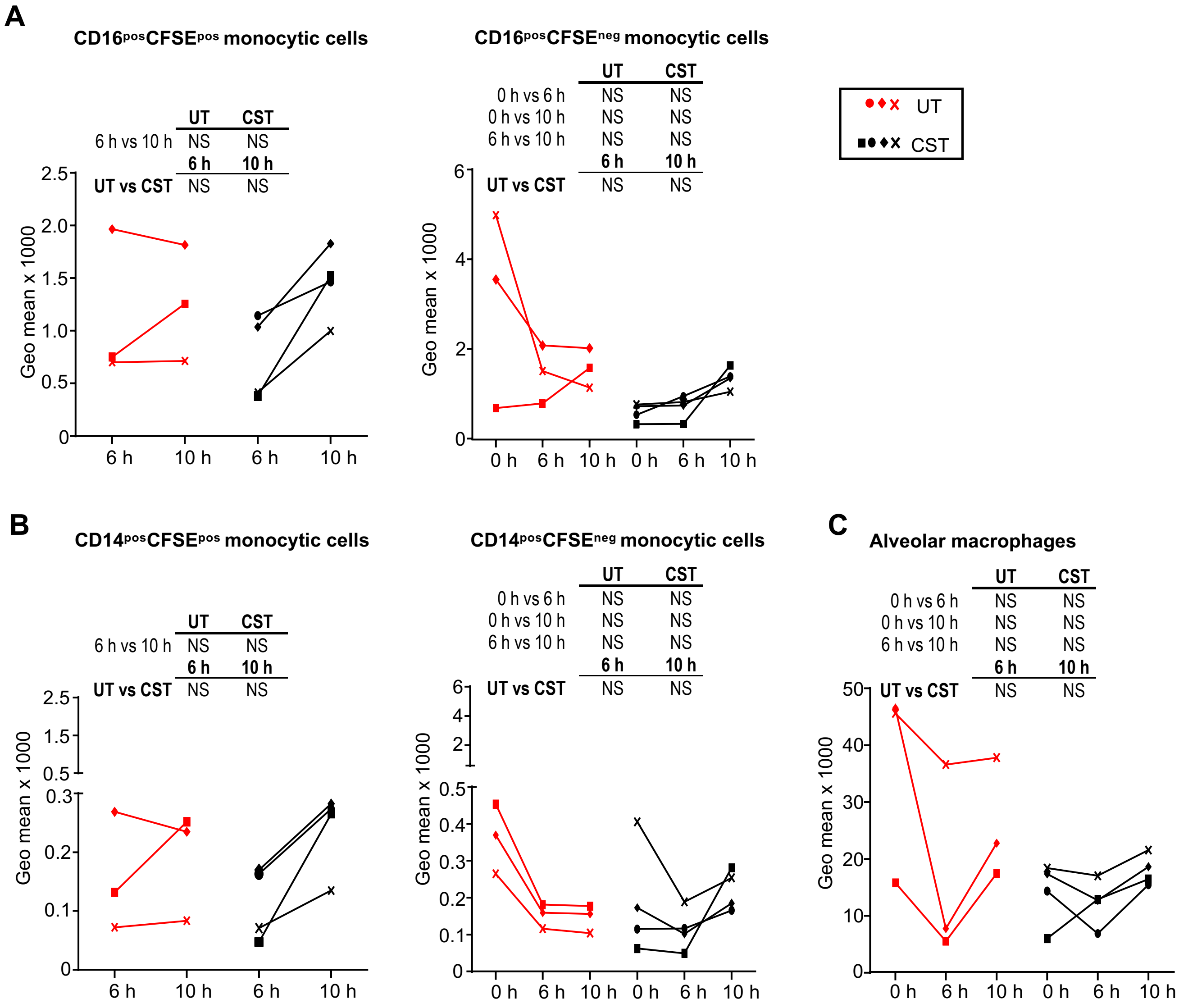

Supplemental Figure 11. Surface expression of the anti-inflammatory CD163 molecule on lung $CD14^{pos}$ and $CD16^{pos}$ monocytic subsets and AMs upon cross-circulation and effects of CS treatment. A. The geometric mean expression of CD163 was measured on the $CD16^{pos}$ subset of the live $CFSE^{pos}SSC\text{-}A^{lo}CD172^{hi}$ cells (left panel) and of the live $CFSE^{neg}SSC\text{-}A^{lo}CD172^{hi}$ cells (right panel). **B.** Same as in A for the $CD14^{pos}$ subset. **C.** The geometric mean expression of CD163 was measured on alveolar macrophages. Each pig is labelled with a unique colored symbol throughout the paper (untreated group (UT) and corticosteroid-treated (CST), UT in red, CST in black). As the data did not follow a normal distribution, a paired Wilcoxon test was performed to compare values across timing (0 h, 6 h, 10 h) and a one-tailed Mann-Whitney test was performed to compare values across groups (UT vs CST). NS stands for non significant.

Pig number (UT and CST groups)	Warm ischemia (min)	Cold storage (min)
UT1	95	105
UT2	84	95
UT3	88	105
UT4	81	115
UT5	92	81
UT Mean ± sd	88 ± 5.7	107.6 ± 18.6
CST1	87	98
CST2	87	98
CST3	77	107
CST4	87	95
CST5	60	140
CST Mean ± sd	79.6 ± 11.8	100.2 ± 12.85

Supplemental Table 1. Ischemic durations (warm and cold) of the donor lungs in the UT and CST groups, showing individual values, and mean ± sd. As the data did not pass the normality test, a bilateral Mann Whitney test was performed to compare the UT and CST values and revealed no statistically significant differences.

Supplemental Table 2. Abs (primary mAbs and secondary Abs) used in the study

Primary antibodies	Provider	Catalog number	Identity[1]	Isotype (murine)	Concentration of use (µg/ml or dilution)
Anti-sw[2]CD13	INRA	in-house production	T35	IgG1	1/100
Anti-swMHCII	WSU	PG2006	MSA3	IgG2a	2 µg/ml
Anti-swCD172A	WSU	PG2031	74.22.15A	IgG2b	2 µg/ml
Anti-huCD80/86	WSU	ANC-501-020	CTLA4-muIg[1]	IgG2a	5 µg/ml
Anti-swCD16	BIO-RAD	MCA1971GA	G7	IgG1	1/100
Anti-huCD14	Thermo-Fisher	MHC400	TUK-4	IgG2a	1/33
Anti-swCD8a	WSU	PG2018	PT81B	IgG2b	2 µg/ml
Anti-swCD335	BIO-RAD	MCA5972GA	VIVKM1	IgG1	2 µg/ml
Anti-swCD4	WSU	PG2013	PT90A	IgG2a	2 µg/ml
Anti-swgranulocyte	WSU	PG2045	PG68A	IgG1	2 µg/ml
Anti-huCD21	BD-Biosciences	555421	B-Ly4	IgG1	5 µg/ml
**Conjugated primary antibodies**	**Provider**	**Catalog number**	**Identity[1]**	**Isotype (murine)**	**Concentration of use (µg/ml or dil)**
Anti-swCD163-PE	BIO-RAD	mca2311pe	2A10/11	IgG1	1/20
Anti-swCD3-PE	BIO-RAD	MCA5951PE	PPT3	IgG1	1/10
Anti-swMHCII-A647	BIO-RAD	MCA2314A6 47	2^{E}9/13	IgG2b	5 µg/ml
Anti-swCD172A-PE	BD Bioscience	561499	74.22.15A	IgG2b	5 µg/ml
Anti-huCD80/86-PE	Ancell	501-050	CTLA4-muIg[1]	IgG2a	5 µg/ml
ISC-G1-PE	Invitrogen	12-4714-82	P3.6.2.8.1	IgG1	1/10
ISC-G2a-PE	BD Bioscience	553930	R35-95	IgG2a	5 µg/ml
ISC-G2b-A647	Biolegend	400626	RTK4530	IgG22	5 µg/ml
**Secondary Antibodies**	**Provider**	**Catalog number**	**Identity[1]**		**Concentration of use (dil)**
Goat anti-mu IgG2b-APC-Cy7	Abcam	ab130791	Goat polyclonal IgG		1/100
Rat anti-mu IgG1-PerCP-eFluor710-Cy5.5	Fisher	15361310	Rat mAb M1-14D12		1/200
Goat anti-mu IgG2a-A647	Invitrogen	A-21241	Goat polyclonal IgG		1/200

[1] Identity corresponds either to the original clone (mAb), a fusion protein (CTLA4-muIg, i.e. human CTLA4 fused to murine IgG2a sequences), or a polyclonal IgG (species of origin). [2]The species of the targeted molecule is either swine (sw), murine (mu), sheep (sh) or human (hu).

GENE	PRIMERS
RPS24	F: AAGGAACGCAAGAACAGAATGAA<br>R:TTTGCCAGCACCAACGTTG
IL10	F: GAGCCAACTGCAGCTTCCA<br>R: TCAGGACAAATAGCCCACTAGCTT
TNFA	F: TGGTGGTGCCGACAGATG<br>R:CAGCCTTGGCCCCTGAA
IL1B	F: GCCCTGTACCCCAACTGGTA<br>R: CCCAGGAAGACGGGCTTT
CXCL8	F: TCCTGCTTTCTGCAGCTCTCT<br>R:GCACTGGCATCGAAGTTCTG
IL6	F:CTGCTTCTGGTGATGGCTACTG<br>R:GGCATCACCTTTGGCATCTT
CCL2	F: ACAGAAGAGTCACCAGCAGCAA<br>R: GCCCGCGATGGTCTTG

Supplemental Table 3. Primers used in the RT-qPCR analysis. The primers were designed with the primer express v.2 software and validated for their efficacy (> 90%) with escalating dose of reference pig cDNA.

Parameter	Group	Time (h)										
		0	1	2	3	4	5	6	7	8	9	10
**Heart rate**	UT	95±24	92±24	88±13	81±16	90±22	90±20	85±19	92±19	91±5	95±11	100±14
**(bpm)**	CST	113±46	106±45	104±46	107±45	85±11	86±7	90±6	84±8	81±15	82±15	96±12
**Systolic BP**	UT	89±9	97±9	94±10	89±11	88±9	93±3	81±12	96±4	100±6	97±8	92±3
**(mm Hg)**	CST	98±20	96±6	103±2	92±14	91±9	89±9	94±13	91±11	90±13	90±13	81±6
**Temperature**	UT	37.6±1.1	37.9±0.8	38.3±0.6	38.5±0.6	38.8±0.6	38.5±0.2	38.5±0.3	38.45±0.3	38.5±0.3	38.5±0.3	38.5±0.4
**(°c)**	CST	37.2±0.5	38.24±0.5	38.5±0.6	38.6±0.5	38.7±0.3	38.6±0.4	38.5±0.3	38.5±0.2	38.5±0.1	38.5±0.1	38.5±0.1
**pH**	UT	7.3±0.04		7.4±0.03		7.5±0.04		7.5±0.04		7.4±0.03		7.4±0.3
	CST	7.3±0.01		7.4±0.05		7.4±0.04		7.4±0.05		7.4±0.06		7.4±0.04
**Lactate**	UT	4.9±3.3		1.5±0.8		1.1±0.7		1.0±0.4		0.9±0.5		0.8±0.2
**(mmol/l)**	CST	3.2±0.8		2.1±1.4		1.4±0.6		1.0±0.2		0.9±0.4		0.7±0.1
**Creatinine**	UT	119±25		120±1		127±14		137±17		148±24		150±26
**(mmol/l)**	CST	102±2		131±13		131±23		131±33		140±33		145±43
**Haemoglobin**	UT	8.9±1.6		6.7±0.4		6.9±0.2		7.3±1.5		8.2±1.5		6.8±0.6
**(g/dl)**	CST	9.2±0.6		7.9±2.0		8.6±2.7		9.2±3.3		8.3±2.3		8.5±2.9
**Glucose**	UT	7.1±2.2		5.8±0.3		6.2±1.2		6.7±1.3		6.6±1.7		5.9±1.6
**(mmol/l)**	CST	6.15±0.7		6.7±1.3		5.4±3.2		7.5±2.1		5.9±1.2		7.3±2.4
**Activated clotting time**	UT	168±32		171±16		177±11		166±19		156±25		174±15
**(s)**	CST	191±26		190±16		173±16		181±8		182±21		185±21
**White Blood Cells**	UT	18.2±2.8	12.9±2.4		14.3±2.3			14.3±2.3				16.8±5.8
**(10^3/mm^3)**	CST	17.6±5.2	14.3±2.4		18.6±4			18.7±4				22.5±2.9
**Lymphocytes**	UT	9.6±1.4	7.3±1.9		6.5±1.2			4.8±0.6				5.3±0.5
**(10^3/mm^3)**	CST	10.6±2.7	7.1±1.3		5.1±0.6			4.9±1.1				5.4±0.9
**Neutrophils**	UT	7.6±2.9	4.3±0.8		7±2.8			9.9±6				10.1±5
**(10^3/mm^3)**	CST	5.9±2.3	6.3±1.7		12.6±3.6			15.4±3.6				15.6±1.8
**Eosinophils**	UT	0.19±0.1	0.11±0.04		0.11±0.04			0.07±0.04				0.10±0.05
**(10^3/mm^3)**	CST	0.3±0.3	0.5±0.8		0.14±0.07			0.09±0.03				0.09±0.05
**Basophils**	UT	0.09±0.09	0.04±0.02		0.08±0.04			0.11±0.05				0.05±0.07
**(10^3/mm^3)**	CST	0.04±0.02	0.07±0.07		0.08±0.05			0.06±0.04				0.06±0.04
**Monocytes**	UT	0.76±0.09	0.54±0.12		0.75±0.12			0.6±0.07				1.2±0.6
**(10^3/mm^3)**	CST	0.78±0.16	0.55±0.09		0.85±0.08			0.87±0.67				1.4±0.47
**Red Blood Cells**	UT	6.6±0.6	4.5±0.6		4.4±0.4			4.3±0.6				4.2±0.6
**(10^6/mm^3)**	CST	6.1±0.5	5.6±0.7		5.3±0.7			5.1±0.5				5±0.6
**Platelets**	UT	323±49	237±90		209±70			204±88				190±60
**(10^3/mm^3)**	CST	304±64	259±57		231±56			224±75				210±45
**Hematocrit**	UT	35.3±5.5	23.8±4.4		22.8±2.4			22.1±2.7				22±3.1
**(%)**	CST	32.9±3.5	30.1±4.1		28.2±4.1			26.8±3.8				26.1±3.8

Supplemental Table 4: Vital and biological parameters in perfusing pigs throughout 10 h of cross-circulation support. The monitored parameters are reported for the UT (red) and CST pig groups (black), 5 pigs/group for vital parameters and 4 pigs/group for blood cell counts. Values represent means ± standard deviations. As the data generally did not pass a normality test, the values from both groups were compared with a two-tailed Mann-Whitney test and revealed no difference between groups.

BP, blood pressure.
